# Delphi consensus guidelines for the use of striatal dopaminergic imaging and cardiac metaiodobenzylguanidine (MIBG) scintigraphy for the diagnosis of dementia and mild cognitive impairment with Lewy bodies

**DOI:** 10.1002/dad2.70296

**Published:** 2026-03-04

**Authors:** Paul C. Donaghy, Gemma Greenfinch, George Petrides, Joseph Kane, Hein J. Verberne, John‐Paul Taylor, John T. O'Brien, Alan J. Thomas, DLB Indicative Imaging Biomarker Study Group, Afina W. Lemstra, Alp Notghi, Andrea Bozoki, Aurelien Lathuiliere, Bhavana Patel, Burc Cagri Poyraz, Carla Abdelnour, Daniel Alcolea, Daniel Weintraub, Douglas Galasko, Durval C. Costa, Federico Massa, Federico Rodriguez‐Porcel, Frederic Blanc, Iosif A. Mendichovszky, Jan Booij, Jan Laczó, Jon B. Toledo, Joseph P. M. Kane, Kathryn Wyman‐Chick, Katrina Cockburn, Kejal Kantarci, Leonidas Chouliaras, Ludy Shih, Manabu Ikeda, Mario Ricciardi, Melissa J. Armstrong, Melissa M. Yu, Mitsuhiro Yoshita, Natasha E. Fullerton, Kevin M. Bradley, Rimona S. Weil, Salvatore Spina, Samantha Holden, Sarah B. Berman, Sonja W. Scholz, Yuto Satake

**Affiliations:** ^1^ Translational and Clinical Research Institute and NIHR Newcastle Biomedical Research Centre Newcastle University Campus for Ageing and Vitality Newcastle upon Tyne UK; ^2^ Institute of Nuclear Medicine University College London Hospitals London UK; ^3^ Department of Nuclear Medicine Newcastle upon Tyne Hospitals NHS Foundation Trust Newcastle Upon Tyne UK; ^4^ Centre for Public Health Queen's University Belfast Institute of Clinical Sciences Royal Victoria Hospital Belfast UK; ^5^ Department of Radiology and Nuclear Medicine Amsterdam UMC Amsterdam the Netherlands; ^6^ Department of Old Age Psychiatry School of Clinical Medicine Cambridge University Cambridge Biomedical Campus Cambridge UK; ^7^ Amsterdam University Medical Centre, Netherlands; ^8^ Sandwell and West Birmingham NHS Trust, UK; ^9^ University of North Carolina Chapel Hill, USA; ^10^ University of Geneva, Switzerland; ^11^ University of Florida College of Medicine, USA; ^12^ Cerrahpasa Medical School, University of Istanbul‐Cerrahpasa, Turkey; ^13^ Department of Neurology and Neurological Sciences, Stanford University School of Medicine, Stanford, USA; ^14^ Hospital de Sant Pau, Spain; ^15^ University of Pennsylvania, USA; ^16^ UC San Diego, USA; ^17^ Champalimaud Foundation, Portugal; ^18^ University of Genoa and IRCCS Ospedale Policlinico San Martino, Genoa, Italy; ^19^ Medical University of South Carolina, USA; ^20^ Hôpitaux Universitaires de Strasbourg, France; ^21^ Department of Radiology, Cambridge University Hospitals NHS Foundation Trust and University of Cambridge, Cambridge, UK; ^22^ Amsterdam University Medical Centers, location University of Amsterdam, Amsterdam, The Netherlands; ^23^ Charles University, Second Faculty of Medicine, Czech Republic; ^24^ Houston Methodist Neurological Institute, USA; ^25^ Queen's University Belfast, UK; ^26^ HealthPartners, USA; ^27^ County Durham and Darlington NHS Trust, UK; ^28^ Mayo Clinic, USA; ^29^ University of Cambridge, UK; ^30^ Beth Israel Deaconess Medical Center, USA; ^31^ Osaka University, Japan; ^32^ Fleni, Argentina; ^33^ Baylor College of Medicine, USA; ^34^ Hokuriku National Hospital, Japan; ^35^ Queen Elizabeth University Hospital, Glasgow, UK; ^36^ Cardiff University, UK; ^37^ University College London, UK; ^38^ University of California San Francisco, USA; ^39^ University of Colorado, USA; ^40^ University of Pittsburgh, USA; ^41^ National Institutes of Health, USA

**Keywords:** dementia with Lewy bodies, imaging, ioflupane, metaiodobenzylguanidine, mild cognitive impairment with Lewy bodies

## Abstract

**INTRODUCTION:**

The aim of this Delphi process was to develop consensus guidelines to support the effective use of striatal dopaminergic imaging and cardiac metaiodobenzylguanidine (MIBG) scintigraphy in the diagnosis of dementia with Lewy bodies and mild cognitive impairment with Lewy bodies.

**METHODS:**

A Delphi consensus panel of 37 international experts independently indicated their agreement or disagreement with statements on indications for the use of these biomarkers and clinical situations in which each biomarker should and should not be used. Statements were accepted if they reached > 80% agreement.

**RESULTS:**

Overall, 36/70 statements (51%) were accepted in Round 1, and 19/37 (51%) were accepted in Round 2.

**DISCUSSION:**

The Delphi consensus process has developed freely available guidelines outlining clinical situations in which striatal dopaminergic imaging and cardiac MIBG scintigraphy may be useful and considerations for their use, including the effect of specific comorbidities and medications on each of the biomarkers.

## Highlights

- Striatal dopaminergic imaging and cardiac metaiodobenzylguanidine scintigraphy are indicative biomarkers for dementia with Lewy bodies.
- The application of these biomarkers in clinical practice remains variable.
- A Delphi process was carried out with an international panel of 37 experts.
- Consensus was gained on how and when to use these biomarkers.
- The resulting guidelines can be adapted and used in clinical services.

## 1 INTRODUCTION

Dementia with Lewy bodies (DLB) and its prodrome, mild cognitive impairment with Lewy bodies (MCI‐LB), are common causes of cognitive impairment. DLB and MCI‐LB are under‐recognized clinically, but clinical diagnosis can be supported with imaging biomarkers, including striatal dopaminergic imaging and cardiac metaiodobenzylguanidine ([123I]‐MIBG) scintigraphy (henceforward “cardiac MIBG scintigraphy”). These two imaging biomarkers are included as “indicative biomarkers” in the diagnostic criteria for DLB and “proposed biomarkers” for MCI‐LB.^1^, ^2^

Striatal dopaminergic imaging is most often carried out using tracers with affinity for the dopamine transporter, to detect the loss of nigrostriatal dopaminergic neurones.^3^ Cardiac MIBG scintigraphy uses a noradrenaline analogue to identify the loss of cardiac sympathetic innervation.^4^ The accuracy of these biomarkers has been demonstrated in DLB and, to a lesser extent, MCI‐LB.^5^, ^6^, ^7^, ^8^ However, there are circumstances in which one biomarker may be preferred over the other. For example, striatal dopaminergic imaging may be less accurate in the presence of medications that have affinity for the dopamine transporter, or when comorbidities may affect the nigrostriatal dopaminergic neurons. Similarly, cardiac MIBG may be less accurate in the presence of medications with effects on noradrenaline reuptake and vesicular storage, or in conditions that may affect peripheral autonomic neurons.

The aim of this Delphi process^9^ was to produce clear and simple guidelines to support clinicians in the effective use of striatal dopaminergic imaging and cardiac MIBG scintigraphy. There have been few head‐to‐head trials of striatal dopaminergic imaging and cardiac MIBG scintigraphy, but these biomarkers have now been used for some time in clinical services. Therefore, the use of a Delphi consensus approach was an appropriate method to agree upon best practices among experts in this field.

The guidelines are intended for use when DLB or prodromal DLB are part of the differential diagnosis, in any clinical service in which one or both of these biomarkers are available.

## 2 METHODS

### 2.1 Leadership group and Delphi panel

The Delphi process was led by a core research group (P.C.D., G.G., G.P., J.P.T., H.V., J.O.B., A.J.T.) with specific expertise relating to the clinical assessment of DLB/MCI‐LB, striatal dopaminergic imaging, and cardiac MIBG. International experts in these fields were approached for participation in the Delphi panel. Participation in the Delphi panel was also offered to members of the Lewy Body Dementias Professional Interest Area (PIA) of the Alzheimer's Association International Society to Advance Alzheimer's Research and Treatment (ISTAART).^10^ Potential participants were approached via e‐mail and gave their opinion on the Delphi statements via an online form. All participants provided informed consent by completing an online consent form.

### 2.2 Ethics

This project was approved by the Newcastle University Ethics Committee (Ref: 36500/2023).

### 2.3 Literature review

Literature searches were carried out to identify the diagnostic accuracy of striatal dopaminergic imaging in MCI‐LB and DLB. Searches were also carried out to identify known effects of medications and comorbidities on these biomarkers. The evidence from the literature review was used by the core research group to assemble draft statements. These statements were accompanied by references where appropriate and copies of relevant publications were shared with the Delphi panel.

### 2.4 Delphi process

The initial statements were presented to the Delphi panel online. The online portal was trialed by two members of the core research group, but their results were not included in the analysis. For each statement, the panel member could select “agree,” “disagree,” or “unable to comment.” Percentage agreement was calculated as: $\frac{n\;`{\mathrm{Agree}}^{\prime }}{n\;`{\mathrm{Agree}}^{\prime }+\;n\;`{\mathrm{Disagree}}^{\prime }}\times \;100\%$.

Participants who answered “unable to comment” were not included in the calculation of % agreement. Statements with ≥ 80% agreement were accepted. Participants could comment on each statement and propose changes. These responses were reviewed by the core research group prior to the reformulation of statements for Round 2. Items agreed in Round 1 were not included in Round 2, unless an alteration had been proposed that might garner greater agreement. When the statement in Round 2 did not reach 80% agreement, the accepted statement in Round 1 was used.

After each round, the results were fed back to the panel. Panelist responses remained anonymous except to the lead author (P.C.D.).

### 2.5 Strength of recommendation

The majority of the recommendations are based on expert consensus, with limited direct evidence. However, there has been a significant amount of research investigating the sensitivity and specificity of these biomarkers, particularly for the diagnosis of DLB. Therefore, a strength of recommendation was assigned by the authors, based on the Strength of Recommendation Taxonomy.^11^ This allows the categorization of strength of recommendation into three categories:

- Consistent, good quality patient‐oriented evidence.
- Inconsistent or limited quality patient‐oriented evidence.
- Consensus, disease‐oriented evidence, usual practice, expert opinion, or case series for studies of diagnosis, treatment, prevention, or screening.

## 3 RESULTS

Round 1 of the Delphi took place from March 13, 2024 to April 29, 2024. Round 2 of the Delphi took place from July 1, 2024 to September 11, 2024. Overall, 37 panelists completed Round 1 and 35 panelists completed Round 2 (see Appendix for panel members). A total of 41% of the panel were female; 43% were from North America, 41% from Europe, 11% from Asia, and 5% from South America; 57% were neurologists, 19% radiologists or nuclear medicine physicians, 16% psychiatrists, and 3% each were geriatricians, medical physicists, and psychologists.

Of the 70 statements, 36 (51%) were accepted in Round 1, and 19/37 (51%) were accepted in Round 2. Supplementary Documents S1 and S2 in supporting information list the questions and % agreement for each statement in Rounds 1 and 2.

In Round 1, the use of striatal dopaminergic imaging and cardiac MIBG scintigraphy when DLB or MCI‐LB is suspected was agreed. The panel did not agree that the biomarkers should only be used in “possible DLB” and not “probable DLB”; did not agree that the biomarkers are indicated when only supportive clinical features are present, without core features; and did not agree that the biomarkers should be used in rapid eye movement (REM) sleep behavior disorder (RBD) without MCI.

**RESEARCH IN CONTEXT**

1. **Systematic review**: Literature searches were carried out to identify the diagnostic accuracy of striatal dopaminergic imaging and cardiac metaiodobenzylguanidine (MIBG) scintigraphy in mild cognitive impairment and dementia with Lewy bodies. Searches were also carried out to identify known effects of medications and comorbidities on these biomarkers. Key papers were shared with the Delphi panel.
2. **Interpretation**: The outcome of the Delphi combines information from the systematic review and the expertise of a wide range of professionals to form a simple guideline that will support the effective use of the biomarkers in clinical practice.
3. **Future directions**: The Delphi consensus guideline is available under a Creative Commons credit to the creator (CC‐BY) license and so can be adapted and used in clinical services. Further research is needed to inform areas in which consensus was not possible, specifically the amount of time that should elapse before repeat dopaminergic imaging or cardiac MIBG is undertaken in people with an initial normal scan.

Choice of scan was agreed in Round 2 after a revision of the phrasing of the statement. Several clinical situations when striatal dopaminergic imaging or cardiac MIBG scintigraphy are particularly useful, or should not be used, were agreed in Round 1. Others were agreed in Round 2 after reframing the statements based on panel feedback.

There was no consensus on when repeat imaging in the same modality should be considered. Only 3% of the panel thought that repeat striatal dopaminergic imaging should not be undertaken at all and 19% thought repeat cardiac MIBG scintigraphy should not be undertaken at all. Despite this, the panel did not agree that repeat imaging could be considered after 24 months, agreeing only that repeat imaging should only be undertaken if there has been significant clinical progression and the diagnosis remains uncertain.

In two instances, multiple related statements were revised for Round 2 and received partial agreement (some were agreed but some were not). To preserve the clarity and consistency of the document, results for Round 1 were used, with a footnote made in the guideline (see Supplementary Documents S3 in supporting information).

The accepted statements are shown in Table 1. The statements were combined when appropriate to improve concision and typographical errors were corrected for the final guideline (Supplementary Documents S4 in supporting information).

**TABLE 1:** Delphi accepted statements after Rounds 1 and 2 with % agreement.

**1. Indication for use**	
Striatal dopaminergic imaging and cardiac MIBG scintigraphy should only be used where the result will have an impact on patient care or quality of life	89%
Striatal dopaminergic imaging is indicated in the following situations:	
In cases where the diagnosis is uncertain, and DLB is suspected.	100%
In cases where the diagnosis is uncertain, and MCI‐LB is suspected.	92%
In late onset (> 60 years) psychiatric disorders (without MCI) where Lewy body disease is a suspected cause.	94%
In recurrent, prolonged, or unexplained delirium (without MCI), where Lewy body disease is a suspected cause.	94%
Cardiac MIBG scintigraphy is indicated in the following situations:	
In cases where the diagnosis is uncertain, and DLB is suspected.	87%
In cases where the diagnosis is uncertain, and MCI‐LB is suspected.	83%
**2. Choice of scan**	
When striatal dopaminergic imaging and cardiac MIBG are both available, dopaminergic imaging should be the first choice investigation in most cases.	85%
**3. Striatal dopaminergic imaging**	
Striatal dopaminergic imaging is particularly useful in cases where parkinsonism is suspected, but not certain clinically.	94%
Striatal dopaminergic imaging is particularly useful to differentiate parkinsonism due to DLB/MCI‐LB from drug induced parkinsonism.	97%
Striatal dopaminergic imaging should be interpreted alongside recent structural imaging (i.e., CT or MRI).	89%
Striatal dopaminergic imaging may be useful to differentiate parkinsonism due to DLB/MCI‐LB from vascular parkinsonism.	91%
Striatal dopaminergic imaging may be abnormal in frontotemporal dementia.	84%
Striatal dopaminergic imaging should not be used to differentiate DLB/MCI‐LB from progressive supranuclear palsy, corticobasal syndrome, or multisystem atrophy.	86%
The following sections refer specifically to [123]I‐FP‐CIT SPECT, the most widely used ligand for striatal dopamine transporter imaging. The following medications and recreational drugs should be stopped for five half‐lives before undertaking striatal dopaminergic imaging using [123]I‐FP‐CIT SPECT:	
Cocaine	100%
Amphetamines	100%
Methylphenidate	100%
Modafinil	97%
Buproprion	91%
Radafaxine	85%
Ephedrine and phenteramine	94%
Consideration should be given to stopping the following medications prior to imaging and if they are not stopped, they should be taken into account when interpreting [123]I‐FP‐CIT SPECT.	
Fentanyl	93%
Codeine	86%
Ketamine, phencyclidine, isofluorane	93%
The use of the following drugs should be noted when interpreting [123]I‐FP‐CIT SPECT:	
Lithium (may decrease signal)	89%
Selective serotonin reuptake inhibitors (may increase signal)	91%
Drugs should only be stopped in consultation with the patient and their clinical team, with consideration of the risks of temporarily stopping medications the likelihood of the medication significantly affecting the scan result.	100%
Dopaminergic imaging abnormalities caused by medications would be expected to cause balanced loss. Evidence of regional loss (e.g., in one putamen) is more suggestive of striatal dopamine transporter loss associated with neurodegeneration.	91%
**4. Cardiac MIBG**	
Cardiac MIBG may be particularly useful:	
When the differential diagnosis includes progressive supranuclear palsy, corticobasal syndrome, or multisystem atrophy.	80%
In patients that are unable to complete SPECT imaging of the head (e.g., because of claustrophobia or inability to keep head still).	91%
In patients with an indeterminate/borderline result on striatal dopaminergic imaging.	94%
Cardiac MIBG scintigraphy should not be used in people with:	
Heart failure – New York Heart Classification Class II and above (mild shortness of breath and/or angina and slight limitation during ordinary activity).	86%
Autonomic neuropathy (including diabetic autonomic neuropathy).	90%
Diabetes with end organ damage (e.g., retinopathy, nephropathy, peripheral neuropathy).	93%
A history of recent myocardial infarction (past 12 months).	93%
When using cardiac MIBG scintigraphy, conclusions about the presence of Lewy body disease should be made with caution in people with:	
A history of myocardial infarction	96%
Diabetes	85%
For patients with diabetes, information of the duration of diabetes, severity, and disease control (including medication taken) should be considered by those requesting and interpreting the MIBG scintigraphy result.	97%
The following medications and recreational drugs should be stopped for five half‐lives before undertaking cardiac MIBG scintigraphy as they may reduce the heart:mediastinum ratio:	
Labetalol	96%
Reserpine/guanethidine/bretylium	100%
Tricyclic antidepressants	96%
Sympathomimetics and decongestants (e.g., phenylpropanolamine, ephedrine, pseudoephedrine, phenylephrine, isoproterenol, terbutaline, fenoterol, xylometazoline)	92%
Cocaine, amphetamine	100%
Methylphenidate	95%
Consideration should be given to stopping the following medications prior to imaging. If they are not stopped, they should be taken into account when interpreting cardiac MIBG imaging	
Noradrenaline and serotonin/noradrenaline reuptake inhibitors (SNRIs)	81%
Tramadol, methadone, pethidine, dextropmethorphan, fentanyl, tapentadol (EANMEANC Guidelines [Flotats 2010] recommend stopping all opiates, but opiates not on this list have low affinity for NET [Rickli 2018])	91%
EANM/EANC guidelines recommend stopping first generation antipsychotics prior to imaging. There is uncertainty about their effect on cardiac MIBG, but first generation antipsychotics should generally be avoided in people with suspected MCI‐LB/DLB	93%
**5. Multiple different types of scans**	
If striatal dopaminergic imaging is normal, but DLB is still suspected, cardiac MIBG scintigraphy is an appropriate investigation.	82%
If cardiac MIBG scintigraphy is normal, but DLB is still suspected, striatal dopaminergic imaging is an appropriate investigation.	81%
If striatal dopaminergic imaging is normal, but MCI‐LB is still suspected, cardiac MIBG scintigraphy is an appropriate investigation.	82%
If cardiac MIBG scintigraphy is normal, but MCI‐LB is still suspected, striatal dopaminergic imaging is an appropriate investigation.	88%
**6. Repeat scans in the same modality**	
If striatal dopaminergic imaging is normal, repeat striatal dopaminergic imaging should only be undertaken if there has been significant clinical progression and the diagnosis remains uncertain.	94%
If cardiac MIBG scintigraphy is normal, further cardiac MIBG scintigraphy should only be undertaken if there has been significant clinical progression and the diagnosis remains uncertain.	83%

Abbreviations: CT, computed tomography; DLB, dementia with Lewy bodies; FP‐CIT, N‐ω‐fluoropropyl‐2β‐carbomethoxy‐3β‐4‐iodophenyl nortropane; MRI, magnetic resonance imaging; MCI‐LB, mild cognitive impairment with Lewy bodies; MIBG, metaiodobenzylguanidine; SPECT, single‐photon emission computed tomography.

## 4 DISCUSSION

This Delphi consensus process has established clear and concise guidelines that will support clinicians in the effective use of striatal dopaminergic imaging and cardiac MIBG scintigraphy in the diagnosis of MCI‐LB and DLB. An important feature of the final guideline is that it is freely available online (via the supporting information in this publication) and can be adapted to meet the needs of different clinical services, for example, when only one of the imaging modalities is available.

### 4.1 Indications

The panel agreed that striatal dopaminergic imaging and cardiac MIBG scintigraphy are both indicated in the investigation of DLB or MCI‐LB, consistent with current diagnostic criteria. Strength of recommendation was assigned using strength of recommendation taxonomy (SORT).^11^ The recommendation for the use of both biomarkers was rated as “A” for DLB, based on multiple case–control/cohort studies and *post mortem* validation of both biomarkers.^8^, ^12^, ^13^, ^14^ The recommendation for MCI‐LB was rated as “B” for both biomarkers because fewer patients with MCI‐LB have been imaged using these biomarkers, the sensitivity of the biomarkers appears to be lower at this stage of disease, and *post mortem* validation is not yet available.^15^

The panel agreed that striatal dopaminergic imaging is also indicated in late onset psychiatric disorders and recurrent, prolonged, or unexplained delirium (without MCI), when Lewy body disease is a suspected cause. Cardiac MIBG scintigraphy did not reach consensus for these indications. The reason for this difference is unclear, as there is limited evidence for either biomarker in these clinical situations, but may reflect a greater degree of clinical experience with striatal dopaminergic imaging. Due to the limited evidence available, these indications were rated as “C” for strength of recommendation. The Delphi consensus panel did not agree that RBD without MCI or dementia was an indication for the use of striatal dopaminergic imaging or cardiac MIBG scintigraphy (< 25% agreement for both biomarkers). While the guidelines did not recommend the use of these imaging biomarkers in clinical practice, this by no means suggests that research using these biomarkers in RBD should not take place. On the contrary, further research is needed to understand the prognostic implications of positive imaging biomarkers in RBD.

When both imaging biomarkers were available, the panel agreed that striatal dopaminergic imaging should be the first line in most cases. There have been few head‐to‐head studies of striatal dopaminergic imaging and cardiac MIBG scintigraphy, and the results have been inconsistent.^5^, ^16^, ^17^, ^18^, ^19^, ^20^ For this reason, the strength of recommendation was rated as “C.” The preference of the panel for dopaminergic imaging may reflect greater experience with this biomarker and the presence of fewer issues with common comorbidities (e.g., diabetes) and medications compared to cardiac MIBG.

Throughout the rest of the guideline, the majority of recommendations were based on expert opinion, case‐series, or indirect evidence (e.g., knowledge of the pharmacodynamic properties of medications). As such, the strength of recommendation has been rated as “C” in most cases. When the strength of recommendation was higher than “C,” the evidence for the recommendation is set out below.

### 4.2 Striatal dopaminergic imaging

In the diagnostic criteria for DLB and MCI‐LB, one cardinal feature of parkinsonism is sufficient to endorse the core clinical feature of parkinsonism (bradykinesia, rest tremor or rigidity).^1^, ^2^ The Delphi panel agreed that striatal dopaminergic imaging may be particularly useful in cases in which parkinsonism is suspected, but not certain, clinically.^1^, ^2^ This may include cases in which the clinician is uncertain whether their patient has benign essential tremor or parkinsonism, or cases in which the clinical assessment was limited due to comorbid conditions such as arthritis.

The guideline highlights that striatal dopaminergic imaging may be abnormal in frontotemporal dementia (SORT B).^21^, ^22^

The panel agreed that striatal dopaminergic imaging should not be used to differentiate DLB or MCI‐LB from progressive supranuclear palsy, corticobasal syndrome, or multiple system atrophy, as there is good evidence of striatal dopaminergic imaging abnormalities in these conditions (SORT A).^23^ The panel agreed that striatal dopaminergic imaging may be useful to differentiate DLB/MCI‐LB from drug‐induced or vascular parkinsonism. The evidence for this recommendation comes from studies reporting relatively small numbers of cases with drug‐induced or vascular parkinsonism (SORT B).^24^

### 4.3 Cardiac MIBG scintigraphy

The panel agreed that striatal dopaminergic imaging should not be used to differentiate DLB or MCI‐LB from progressive supranuclear palsy, corticobasal syndrome, or multiple system atrophy. Conversely, the panel agreed that cardiac MIBG scintigraphy may be particularly useful when the differential diagnosis includes progressive supranuclear palsy, corticobasal syndrome, or multiple system atrophy.^25^, ^26^ However, some panel members highlighted that abnormal cardiac MIBG scans have been reported in patients with clinically diagnosed progressive supranuclear palsy and multiple system atrophy.^27^ The nature of these apparent false positive results is unclear. They may represent true “false positives,” or they could reflect cases of Lewy body disease that meet clinical criteria for progressive supranuclear palsy or corticobasal syndrome.

The Delphi panel recommended that cardiac MIBG scintigraphy should not be used to help diagnose DLB or MCI‐LB in people with heart failure, autonomic neuropathy, diabetes with end organ damage, or a history of recent myocardial infarction. Cardiac MIBG may be abnormal in these conditions, reducing the specificity of the scan for DLB. It recommended that conclusions about the presence of Lewy body disease should be made with caution in people with a history of myocardial infarction or diabetes. Large trials have demonstrated the presence of abnormal cardiac MIBG imaging in heart failure (SORT A).^28^ The remaining recommendations are based on smaller cohorts or more indirect evidence (SORT B).^29^

### 4.4 Medications

Previously published recommendations and reviews on the effect of medications on dopaminergic imaging and cardiac MIBG scintigraphy have given inconsistent guidance.^3^, ^4^, ^30^, ^31^ The Delphi panel generally took a less restrictive approach to imaging while patients are taking medications which have a theoretical effect on the imaging results. Where the guideline differed from other recommendations, this was outlined in footnotes in the final guideline.

In several cases, the panel recommended taking the medication into account while interpreting the scan, rather than stopping the medication. This statement recognizes that there are circumstances in which medications may have a minor effect on [123I]N‐ω‐fluoropropyl‐2β‐carbomethoxy‐3β‐4‐iodophenyl nortropane (FP‐CIT) single‐photon emission computed tomography (SPECT) or cardiac MIBG scintigraphy result, but the risks of stopping the medication for five half‐lives prior to the scan are greater than the potential benefits. In these cases, the clinician may have lower certainty in the accuracy of the result, particularly when the result is equivocal (i.e., not clearly normal or markedly abnormal). The clinician should consider this possibility prior to requesting the scan and, as with all imaging, these biomarkers should only be used when the result will have an impact on patient care or quality of life.

### 4.5 Repeat imaging

There were some areas in which consensus was not possible, particularly what length of time should elapse before a repeat scan is undertaken in the same modality. Whereas 19% of the panel thought cardiac MIBG scintigraphy should not be repeated at all, only 3% of the panel felt striatal dopaminergic imaging should not be repeated at all. Despite this, the panel did not agree that repeat striatal dopaminergic imaging could be considered after 24 months. Further research is needed to determine the clinical utility of repeat imaging, with a specific focus on patients with an initial negative scan.

The panel agreed that repeat imaging should only be undertaken if there has been significant clinical progression and the diagnosis remains uncertain. The definition of “significant clinical progression” is not operationalized in the guideline, and it is for individual clinicians to determine whether they consider significant clinical progression to have taken place. The difficulty achieving consensus in this statement may relate to the lack of evidence in this area. As a result, the strength of this recommendation was classed as “C.”

### 4.6 Striatal dopaminergic imaging and cardiac MIBG scintigraphy in the context of new diagnostic systems

While these guidelines are intended to support clinicians when DLB or MCI‐LB are part of the differential diagnosis, new diagnostic systems have been proposed that take a transdiagnostic approach to synucleinopathies. The advent of synuclein biomarkers, such as seed amplification assays, may have been expected to diminish the importance of striatal dopaminergic imaging and cardiac MIBG scintigraphy. However, both biomarkers are included as markers of neurodegeneration in the SynNeurGe research diagnostic criteria for Parkinson's disease^32^ and “dopamine dysfunction” is a key anchor of the neuronal α‐synuclein disease integrated staging system (NSD‐ISS).^33^ The NSD‐ISS criteria also highlight that other validated markers of “neuronal α‐synuclein disease specific neurodegeneration” could be included in the criteria in future. Cardiac MIBG is a potential candidate for inclusion. These criteria make it clear that striatal dopaminergic imaging and cardiac MIBG scintigraphy are likely to be an important feature in the diagnosis of synucleinopathies for the foreseeable future, and their appropriate use in the investigation of MCI‐LB and DLB, as set out in this guideline, will continue to be an essential part of clinical practice.

### 4.7 Strengths and limitations

The Delphi panel was diverse in terms of geographical region, sex, and clinical specialty, though participants from North America/Europe and neurologists were over‐represented. The membership of the Delphi panel was not limited to people known by the authors, and all members of the Lewy Body Dementia PIA of the Alzheimer's Association ISTAART were invited to participate. Several of the authors have received support from GE Healthcare, but none of these authors participated in the Delphi panel vote.

The guideline specifically aims to support clinicians when the diagnosis of MCI‐LB or DLB is suspected and does not aim to provide guidance for other uses of the biomarkers, for example in the diagnosis of Parkinson's disease.

The panel was asked to agree or disagree with recommendations based on the available evidence and not based on what imaging modalities are available in their region. However, clinical experience with one or other imaging modality may have affected participants’ responses. A total of 33 members of the Delphi panel had access to FP‐CIT SPECT or another form of striatal dopaminergic imaging, whereas 19 had access to MIBG. As 84% of the Delphi panel were from Europe or North America, the consensus may not reflect clinical opinion in Asia, South America, or Oceania. The guidelines are available under a Creative Commons credit to the creator (CC‐BY) license and can be adjusted for regions where only one modality is available.

Some statements that reached agreement in Round 1 were revised for Round 2 based on Delphi participant comments. If the Round 2 statement was not agreed, the Round 1 statement was used. In two cases, the revised statements received partial agreement, leaving guideline statements that would either be overly complicated (three levels of medication advice for FP‐CIT SPECT, in addition to a footnote) or omitted key information (no statement on corticobasal syndrome or multiple system atrophy). In both cases, we reverted to the Round 1 statements to ensure clarity, consistency, and completeness of the guideline. This is set out in more detail in Supplementary Documents S3.

### 4.8 Research priorities

The Delphi process highlighted some areas in which further research is required, including these key areas:

- Autopsy validation of striatal dopaminergic imaging and cardiac MIBG scintigraphy in mild cognitive impairment with Lewy bodies.
- The diagnostic accuracy of striatal dopaminergic imaging and cardiac MIBG scintigraphy in other prodromal presentations of DLB, including psychiatric onset, delirium onset and RBD without MCI.
- Head‐to‐head comparisons of striatal dopaminergic imaging and cardiac MIBG scintigraphy in different clinical presentations.
- The utility of repeat imaging and minimum timeframe before repeat imaging should be considered.

## 5 CONCLUSION

This guideline provides clinicians with a clear, concise tool that will support effective decision making when choosing imaging biomarkers to identify MCI‐LB and DLB.

## CONFLICT OF INTEREST STATEMENT

P.C.D. has received funding from GE Healthcare for an investigator led study involving 123I‐FP‐CIT SPECT. G.G. has received honoraria from GE Healthcare for delivering educational workshops on FP‐CIT imaging. G.P. has received honoraria from GE Healthcare for delivering educational workshops on FP‐CIT imaging, for FP‐CIT clinical reporting, and for contributing to GE Healthcare led studies. J.K. has received honoraria from Eisai for educational presentations on dementia practice and has acted as a consultant for Takeda. H.V. has nothing to report. J.P.T. has received honoraria from GE Healthcare for delivering educational presentations on Lewy body disease. J.O.B. has acted as a consultant for TauRx, Novo Nordisk, Biogen, Roche, Lilly, GE Healthcare, and Okwin and received grants or academic in kind support from Avid/ Lilly, Merck, UCB, and Alliance Medical. A.J.T. has received support for investigator led studies and honoraria from GE Healthcare. Author disclosures are available in the supporting information.

## CONSENT STATEMENT

All participants provided informed consent by completing an online consent form.

## Supporting information



Supporting Information

Supporting Information

Supporting Information

Supporting Information

Supporting Information

## 1

COLLABORATORS IN THE DLB INDICATIVE IMAGING BIOMARKER STUDY GROUP

**Table:**

Afina W. Lemstra	Amsterdam University Medical Centre, Netherlands
Alp Notghi	Sandwell and West Birmingham NHS Trust, UK
Andrea Bozoki	University of North Carolina Chapel Hill, USA
Aurelien Lathuiliere	University of Geneva, Switzerland
Bhavana Patel	University of Florida College of Medicine, USA
Burc Cagri Poyraz	Cerrahpasa Medical School, University of Istanbul‐Cerrahpasa, Turkey
Carla Abdelnour	Department of Neurology and Neurological Sciences, Stanford University School of Medicine, Stanford, USA
Daniel Alcolea	Hospital de Sant Pau, Spain
Daniel Weintraub	University of Pennsylvania, USA
Douglas Galasko	UC San Diego, USA
Durval C. Costa	Champalimaud Foundation, Portugal
Federico Massa	University of Genoa and IRCCS Ospedale Policlinico San Martino, Genoa, Italy
Federico Rodriguez‐Porcel	Medical University of South Carolina, USA
Frederic Blanc	Hôpitaux Universitaires de Strasbourg, France
Iosif A. Mendichovszky	Department of Radiology, Cambridge University Hospitals NHS Foundation Trust and University of Cambridge, Cambridge, UK
Jan Booij	Amsterdam University Medical Centers, location University of Amsterdam, Amsterdam, The Netherlands
Jan Laczó	Charles University, Second Faculty of Medicine, Czech Republic
Jon B. Toledo	Houston Methodist Neurological Institute, USA
Joseph P. M. Kane	Queen's University Belfast, UK
Kathryn Wyman‐Chick	HealthPartners, USA
Katrina Cockburn	County Durham and Darlington NHS Trust, UK
Kejal Kantarci	Mayo Clinic, USA
Leonidas Chouliaras	University of Cambridge, UK
Ludy Shih	Beth Israel Deaconess Medical Center, USA
Manabu Ikeda	Osaka University, Japan
Mario Ricciardi	Fleni, Argentina
Melissa J. Armstrong	University of Florida College of Medicine, USA
Melissa M. Yu	Baylor College of Medicine, USA
Mitsuhiro Yoshita	Hokuriku National Hospital, Japan
Natasha E. Fullerton	Queen Elizabeth University Hospital, Glasgow, UK
Kevin M. Bradley	Cardiff University, UK
Rimona S. Weil	University College London, UK
Salvatore Spina	University of California San Francisco, USA
Samantha Holden	University of Colorado, USA
Sarah B. Berman	University of Pittsburgh, USA
Sonja W. Scholz	National Institutes of Health, USA
Yuto Satake	Osaka University, Japan
