# Supplementary material for: Delphi consensus guidelines for the use of striatal dopaminergic imaging and cardiac metaiodobenzylguanidine (MIBG) scintigraphy for the diagnosis of dementia and mild cognitive impairment with Lewy bodies
Source: Alzheimers Dement (Amst). 2026 Mar 4;18(1):e70296. doi: 10.1002/dad2.70296 (PMC12960062; doi:10.1002/dad2.70296)
Supplement: Supplementary file 1 — Supporting Information [file DAD2-18-e70296-s001.docx]

**Delphi Round 1: Statements and % Agreement**

**1. Indication for use**

| Striatal dopaminergic imaging and cardiac MIBG scintigraphy should only be used where the result will have an impact on patient care or quality of life | 89% |
| --- | --- |

**Striatal dopaminergic imaging is indicated in the following situations:**

| In cases of where the diagnosis is uncertain, and DLB is suspected. | 100% |
| --- | --- |
| Only in patients fulfilling criteria for ‘possible DLB’ and not in those fulfilling criteria for ‘probable DLB’ (McKeith 2017). | 38% |
| In cases where only supportive clinical features of DLB are present and no core features are present | 63% |
| In cases of where the diagnosis is uncertain, and MCI-LB is suspected. | 92% |
| Only in patients fulfilling criteria for ‘possible MCI-LB’ (McKeith 2020) and not in those fulfilling criteria for ‘probable MCI-LB’. | 31% |
| In cases where only supportive clinical features of MCI-LB are present and no core features are present | 58% |

**Cardiac MIBG scintigraphy is indicated in the following situations:**

| In cases of where the diagnosis is uncertain, and DLB is suspected. | 87% |
| --- | --- |
| Only in patients fulfilling criteria for ‘possible DLB’ and not in those fulfilling criteria for ‘probable DLB’ (McKeith 2017). | 41% |
| In cases where only supportive clinical features of DLB are present and no core features are present | 55% |
| In cases of where the diagnosis is uncertain, and MCI-LB is suspected. | 83% |
| Only in patients fulfilling criteria for ‘possible MCI-LB’ (McKeith 2020) and not in those fulfilling criteria for ‘probable MCI-LB’. | 34% |
| In cases where only supportive clinical features of MCI-LB are present and no core features are present | 59% |

**2.** **Choice of scan**

| When striatal dopaminergic imaging and cardiac MIBG are both available, which should generally be used as the first choice investigation | 73% dopaminergic imaging |
| --- | --- |

**3. Striatal dopaminergic imaging**

| Striatal dopaminergic imaging is particularly useful in cases where parkinsonism is suspected, but not certain clinically | 94% |
| --- | --- |
| Striatal dopaminergic imaging is particularly useful to differentiate parkinsonism due to DLB/MCI-LB from drug induced parkinsonism | 97% |
| Striatal dopaminergic imaging is particularly useful to differentiate parkinsonism due to DLB/MCI-LB from vascular parkinsonism | 72% |

| Striatal dopaminergic imaging should be interpreted alongside recent structural imaging (i.e. CT or MRI) | 89% |
| --- | --- |

**Striatal dopaminergic imaging should not be used:**

| To differentiate DLB/MCI-LB from frontotemporal dementia | 52% |
| --- | --- |
| To differentiate DLB/MCI-LB from progressive supranuclear palsy, corticobasal syndrome or multisystem atrophy | 86% |
| In the presence of normal pressure hydrocephalus | 41% |

**The following sections refer specifically to [123]I-FP-CIT SPECT, the most widely used ligand for striatal dopamine transporter imaging.**

**The following medications and recreational drugs should be stopped for five half-lives before undertaking striatal dopaminergic imaging using [123]I-FP-CIT SPECT:**

***NOTE FOR DELPHI PANEL MEMBERS: This list is based on EANM/SNMMI Guideline [Morbelli 2020], the guidelines recommend stopping these medications for 5 half-lives prior to imaging.***

| Cocaine | 100% |
| --- | --- |
| Amphetamines | 100% |
| Cannabidiol | 67% |
| Methylphenidate | 100% |
| Modafinil | 97% |
| Buproprion | 91% |
| Radafaxine | 85% |
| Haloperidol  *NOTE FOR DELPHI PANEL MEMBERS: EANM/SNMMI Guideline [Morbelli 2020] does not mention Haloperidol, but it was identified in a recent systematic review [Chahid 2023].* | 72% |
| Ephedrine and phenteramine | 94% |
| Fentanyl | 75% |
| Codeine  *NOTE FOR DELPHI PANEL MEMBERS: EANM/SNMMI Guideline [Morbelli 2020] does not mention Codeine, but it was identified in a recent systematic review [Chahid 2023].* | 60% |
| Benzatropine | 62% |
| Ketamine, phencyclidine, isofluorane | 75% |

**The use of the following drugs should be noted when interpreting [123]I-FP-CIT SPECT:**

| Lithium (may decrease signal) | 89% |
| --- | --- |
| Selective serotonin reuptake inhibitors (may increase signal) | 91% |

**4. Cardiac MIBG Scintigraphy**

**Cardiac MIBG may be particularly useful:**

| When the differential diagnosis includes frontotemporal dementia | 71% |
| --- | --- |
| When the differential diagnosis includes progressive supranuclear palsy, corticobasal syndrome or multisystem atrophy | 80% |
| In patients that are unable to complete SPECT imaging of the head (e.g. because of claustrophobia or inability to keep head still) | 91% |
| In patients with an indeterminate/borderline result on striatal dopaminergic imaging | 94% |

**Cardiac MIBG Scintigraphy should not be used in people with:**

| Heart failure – New York Heart Classification Class II and above (mild shortness of breath and/or angina and slight limitation during ordinary activity) | 86% |
| --- | --- |
| Autonomic neuropathy (including diabetic autonomic neuropathy) | 90% |
| A history of myocardial infarction | 70% |
| Diabetes with end organ damage (e.g. retinopathy, nephropathy, peripheral neuropathy) | 93% |
| Diabetes | 60% |

**When using cardiac MIBG Scintigraphy, conclusions about the presence of Lewy body disease should be made with caution in people with:**

| A history of myocardial infarction | 96% |
| --- | --- |
| Diabetes with end organ damage (e.g. retinopathy, nephropathy, peripheral neuropathy) | 100% |
| Diabetes | 85% |

**The following medications and recreational drugs should be stopped for five half lives before undertaking cardiac MIBG scintigraphy as they may reduce the heart:mediastinum ratio:**

| Labetalol | 96% |
| --- | --- |
| Reserpine/guanethidine/bretylium | 100% |
| Tricyclic antidepressants | 96% |
| Noradrenaline and serotonin/noradrenaline reuptake inhibitors (SNRIs) | 79% |
| Sympathomimetics and decongestants (e.g. phenylpropanolamine, ephedrine, pseudoephedrine, phenylephrine, isoproterenol, terbutaline,  phenoterol, xylometazoline) | 92% |
| Cocaine, amphetamine | 100% |
| Methylphenidate | 95% |
| Tramadol, methadone, pethidine, dextropmethorphan, Fenatnyl  *NOTE FOR DELPHI PANEL MEMBERS: EANM Guidelines [Flotats 2010] recommend stopping all opiates, but opiates not on this list have low affinity for NET [Rickli 2018]* | 74% |

**Cardiac MIBG Scintigraphy should be interpreted with caution in people taking the following medications, due to uncertainty about their effects on MIBG uptake:**

| First generation antipsychotics  *NOTE FOR DELPHI PANEL MEMBERS: EANM Guidelines [Flotats 2010] recommend stopping first generation antipsychotics, but other reviews state the evidence is uncertain [Jacobson 2015]* | 69% |
| --- | --- |
| Quetiapine  *NOTE FOR DELPHI PANEL MEMBERS: EANM Guidelines [Flotats 2010] do not mention second generation antipsychotics in general or quetiapine specifically, but quetiapine metabolites may block noradrenalin transporters in the brain [Nyberg 2013]* | 44% |
| Beta-agonists (e.g. salbutamol)  *NOTE FOR DELPHI PANEL MEMBERS: EANM Guidelines [Flotats 2010] recommend stopping salbutamol, but other reviews recommend continuing beta-agonists [Jacobson 2015]* | 43% |
| Levodopa  *NOTE FOR DELPHI PANEL MEMBERS: EANM Guidelines [Flotats 2010] recommend stopping levodopa, but evidence for an effect on cardiac MIBG heart:mediastinum ratio is not clear [Kishi 2011]* | 35% |
| Calcium channel blockers (may increase signal)  *NOTE FOR DELPHI PANEL MEMBERS: EANM Guidelines [Flotats 2010] recommend stopping calcium channel blockers, but other reviews recommend continuing these medications [Jacobson 2015]* | 29% |
| Trazodone  *NOTE FOR DELPHI PANEL MEMBERS: EANM Guidelines [Flotats 2010] appear to intend to recommend stopping trazodone (mispelt as ‘Trazolone’), but other reviews state the evidence is uncertain [Jacobson 2015]* | 43% |

**5. Multiple different types of scans**

| If striatal dopaminergic imaging is normal, but DLB is still suspected, cardiac MIBG scintigraphy is an appropriate investigation | 82% |
| --- | --- |
| If cardiac MIBG scintigraphy is normal, but DLB is still suspected, striatal dopaminergic imaging is an appropriate investigation | 81% |

| If striatal dopaminergic imaging is normal, but MCI-LB is still suspected, cardiac MIBG scintigraphy is an appropriate investigation | 82% |
| --- | --- |
| If cardiac MIBG scintigraphy is normal, but MCI-LB is still suspected, striatal dopaminergic imaging is an appropriate investigation | 78% |

**6. Repeat scans in the same modality**

| If striatal dopaminergic imaging is normal, repeat striatal dopaminergic imaging should generally not be undertaken in future | 14% |
| --- | --- |
| If striatal dopaminergic imaging is normal, repeat striatal dopaminergic imaging should only be undertaken if there has been significant clinical progression | 69% |
| Multiple choice: If striatal dopaminergic imaging is normal, further striatal dopaminergic imaging should not be undertaken for at least  12 months (43%)  18 months (32%)  24 months (22%, total 97% 24 months or less)  Should not be undertaken at all (3%) |  |
| If cardiac MIBG scintigraphy is normal, further cardiac MIBG scintigraphy should generally not be undertaken in future | 30% |
| If cardiac MIBG scintigraphy is normal, further cardiac MIBG scintigraphy should only be undertaken if there has been significant clinical progression | 67% |
| Multiple choice: If cardiac MIBG scintigraphy is normal, repeat cardiac MIBG scintigraphy should not be undertaken for at least  12 months (32%  18 months (27%)  24 months (22%, total 81% 24 months or less)  Should not be undertaken at all (19%) |  |
