# Supplementary material for: Delphi consensus guidelines for the use of striatal dopaminergic imaging and cardiac metaiodobenzylguanidine (MIBG) scintigraphy for the diagnosis of dementia and mild cognitive impairment with Lewy bodies
Source: Alzheimers Dement (Amst). 2026 Mar 4;18(1):e70296. doi: 10.1002/dad2.70296 (PMC12960062; doi:10.1002/dad2.70296)
Supplement: Supplementary file 2 — Supporting Information [file DAD2-18-e70296-s005.docx]

**Delphi Round 2: Statements and % Agreement**

**1. Indications for use**

**Striatal dopaminergic imaging is indicated in the following situations:**

| In idiopathic REM sleep behaviour disorder (without MCI) | 24% |
| --- | --- |
| In late onset (>60 years) psychiatric disorders (without MCI) where Lewy body disease is a suspected cause | 94% |
| In recurrent, prolonged or unexplained delirium (without MCI), where Lewy body disease is a suspected cause | 94% |

**Cardiac MIBG scintigraphy is indicated in the following situations:**

| In idiopathic REM sleep behaviour disorder (without MCI) | 23% |
| --- | --- |
| In late onset (>60 years) psychiatric disorders (without MCI) where Lewy body disease is a suspected cause | 73% |
| In recurrent, prolonged or unexplained delirium (without MCI), where Lewy body disease is a suspected cause | 73% |

**2. Choice of scan**

*[Note for Delphi Panel Members: 73% of the panel indicated that dopaminergic imaging should be used as the first choice investigation, if dopaminergic imaging and cardiac MIBG were both available. The new statement aims to reflect this, whilst allowing that there may be circumstances where other biomarkers are selected.]*

| When striatal dopaminergic imaging and cardiac MIBG are both available, dopaminergic imaging should be the first choice investigation in most cases. | 85% |
| --- | --- |

**3. Striatal dopaminergic imaging**

| Striatal dopaminergic imaging may be useful to differentiate parkinsonism due to DLB/MCI-LB from vascular parkinsonism | 91% |
| --- | --- |
| Striatal dopaminergic imaging may be abnormal in frontotemporal dementia *[Note for Delphi panel members: Morgan 2012, doi: 10.1136/jnnp-2012-302577 ]* | 84% |

**The following medications and recreational drugs should be stopped for five half-lives before undertaking striatal dopaminergic imaging using [123]I-FP-CIT SPECT:**

| Sertraline | 41% |
| --- | --- |
| Venlafaxine | 37% |
| Lithium | 33% |

*[Note for Delphi Panel Members: EANM/SNMMI guidelines [Morbelli] and/or a recent systematic review [Chahid] recommend stopping the following drugs for 5 half lives prior to imaging. The majority of Delphi panel members agreed with this, but the level of agreement did not reach consensus.]*

**Consideration should be given to stopping the following medications prior to imaging and if they are not stopped, they should be taken into account when interpreting [123]I-FP-CIT SPECT.**

| Cannabidiol | 76% |
| --- | --- |
| Haloperidol | 79% |
| Fentanyl | 93% |
| Codeine | 86% |
| Benzatropine | 69% |
| Ketamine, phencyclidine, isofluorane | 93% |

| Drugs should only be stopped in consultation with the patient and their clinical team, with consideration of the risks of temporarily stopping medications the likelihood of the medication significantly affecting the scan result | 100% |
| --- | --- |

| Dopaminergic imaging abnormalities caused by medications would be expected to cause balanced loss. Evidence of regional loss (e.g. in one putamen) is more suggestive of striatal dopamine transporter loss associated with neurodegeneration. | 91% |
| --- | --- |

**4. Cardiac MIBG Scintigraphy**

**Cardiac MIBG may be particularly useful:**

| When the differential diagnosis includes frontotemporal dementia, as striatal dopaminergic imaging may be abnormal in people with frontotemporal dementia | 74% |
| --- | --- |
| When the differential diagnosis includes corticobasal syndrome or multisystem atrophy | 71% |

| Cardiac MIBG may be useful when the differential diagnosis includes progressive supranuclear palsy, but abnormalities have been reported in progressive supranuclear palsy  *[Note for Delphi Panel Members: Kamada 2019, Sakuramoto 2020, Catalan 2021*  *http://dx.doi.org/10.1016/j.prdoa.2019.12.002*  *https://doi.org/10.1002/mdc3.13227*  *https://doi.org/10.1016/j.jns.2018.10.019]* | 87% |
| --- | --- |

| Cardiac MIBG Scintigraphy should not be used in people with a history of recent myocardial infarction (past 12 months) | 93% |
| --- | --- |

| For patients with diabetes, information of the duration of diabetes, severity and disease control (including medication taken) should be considered by those requesting and interpreting the MIBG scintigraphy result. | 97% |
| --- | --- |

*[Note for Delphi Panel Members: EANM/EANC guidelines recommend stopping the following drugs for 5 half lives prior to imaging. The majority of Delphi panel members agreed with this, but the level of agreement did not reach the consensus.]*

**Consideration should be given to stopping the following medications prior to imaging. If they are not stopped, they should be taken into account when interpreting cardiac MIBG imaging.**

| Noradrenaline and serotonin/noradrenaline reuptake inhibitors (SNRIs) | 81% |
| --- | --- |
| Tramadol, methadone, pethidine, dextropmethorphan, fenatnyl, tapentadol  (EANMEANC Guidelines [Flotats 2010] recommend stopping all opiates, but opiates not on this list have low affinity for NET [Rickli 2018]) | 91% |

| EANM/EANC guidelines recommend stopping first generation antipsychotics prior to imaging. There is uncertainty about their effect on cardiac MIBG, but first generation antipsychotics should generally be avoided in people with suspected MCI-LB/DLB | 93% |
| --- | --- |

*[Note for Delphi Panel Members: <50% of Delphi Panel Members agreed that Cardiac MIBG Scintigraphy should be interpreted with caution in people taking the following medications. If there is no agreement on the statements below, we will include as a footnote that EANM/EANC guidelines recommend stopping these drugs, but that the panel could not reach consensus on this]*

**EANM/EANC guidelines recommend stopping the following drugs for 5 half lives prior to imaging, but there is uncertainty about their effects on MIBG uptake. Cardiac MIBG scintigraphy can be undertaken whilst patients continue to take the following medication:**

| Beta-agonists (e.g. salbutamol)  *NOTE FOR DELPHI PANEL MEMBERS: EANM Guidelines [Flotats 2010] recommend stopping salbutamol, but other reviews recommend continuing beta-agonists [Jacobson 2015]* | 77% |
| --- | --- |
| Levodopa  *NOTE FOR DELPHI PANEL MEMBERS: EANM Guidelines [Flotats 2010] recommend stopping levodopa, but evidence for an effect on cardiac MIBG heart:mediastinum ratio is not clear [Kishi 2011]* | 64% |
| Calcium channel blockers (may increase signal)  *NOTE FOR DELPHI PANEL MEMBERS: EANM Guidelines [Flotats 2010] recommend stopping calcium channel blockers, but other reviews recommend continuing these medications [Jacobson 2015]* | 68% |
| Trazodone  *NOTE FOR DELPHI PANEL MEMBERS: EANM Guidelines [Flotats 2010] appear to intend to recommend stopping trazodone (mispelt as ‘Trazolone’), but other reviews state the evidence is uncertain [Jacobson 2015]* | 55% |

**5. Multiple different types of scan**

| If cardiac MIBG scintigraphy is normal, but MCI-LB is still suspected, striatal dopaminergic imaging is an appropriate investigation | 88% |
| --- | --- |

**6. Repeat scans in the same modality**

| **I**f striatal dopaminergic imaging is normal, repeat striatal dopaminergic imaging should only be undertaken if there has been significant clinical progression and the diagnosis remains uncertain | 94% |
| --- | --- |
| If cardiac MIBG scintigraphy is normal, further cardiac MIBG scintigraphy should only be undertaken if there has been significant clinical progression and the diagnosis remains uncertain | 83% |
| If striatal dopaminergic imaging is normal, repeat striatal dopaminergic imaging could be considered after 24 months | 70% |
| If cardiac MIBG scintigraphy is normal, repeat cardiac MIBG scintigraphy could be considered after 24 months | 64% |
