# Supplementary material for: Delphi consensus guidelines for the use of striatal dopaminergic imaging and cardiac metaiodobenzylguanidine (MIBG) scintigraphy for the diagnosis of dementia and mild cognitive impairment with Lewy bodies
Source: Alzheimers Dement (Amst). 2026 Mar 4;18(1):e70296. doi: 10.1002/dad2.70296 (PMC12960062; doi:10.1002/dad2.70296)
Supplement: Supplementary file 3 — Supporting Information [file DAD2-18-e70296-s002.docx]

**Note 1**

**Results from Round 1**

The following medications and recreational drugs should be stopped for five half-lives before undertaking striatal dopaminergic imaging using [123]I-FP-CIT SPECT:

|  | **Agreement** |
| --- | --- |
| Cannabidiol | 67% |
| Haloperidol | 72% |
| Fentanyl | 75% |
| Codeine | 60% |
| Benzatropine | 62% |
| Ketamine, phencyclidine, isofluorane | 75% |

**Results from Round 2**

Consideration should be given to stopping the following medications prior to imaging and if they are not stopped, they should be taken into account when interpreting [123]I-FP-CIT SPECT.

|  | **Agreement** |
| --- | --- |
| Cannabidiol | 76% |
| Haloperidol | 79% |
| Fentanyl | 93% |
| Codeine | 86% |
| Benzatropine | 69% |
| Ketamine, phencyclidine, isofluorane | 93% |

**Note**

The statement in round two was revised with an aim to have two statements referring to medications and FP-CIT SEPCT. However, three of the medications reached consensus on this, and three did not. If the statement agreed in Round 2 for Fentanyl/Codeine/Ketamine was included, there would be 3 different levels of advice around medication for FP-CIT SPECT, in addition to a footnote. The Core Group and Delphi Panel agreed that this would make the guidelines over-complicated. Instead, a statement of fact about the Round 1 results was added as a footnote for these medications: “EANM/SNMMI guidelines [Morbelli et al. 2020 doi:10.1007/s00259-020-04817-8] and/or a recent systematic review [Chahid et al. 2023 doi:10.1007/s00259-023-06171-x] recommend stopping the following drugs for 5 half-lives prior to imaging. The majority of Delphi panel members agreed with this, but the level of agreement did not reach consensus: fentanyl, codeine, ketamine, phencyclidine, isoflurane, cannabidiol, haloperidol, benzatropine.”

**Note 2**

**Result from Round 1:**

Cardiac MIBG may be particularly useful:

| When the differential diagnosis includes progressive supranuclear palsy, corticobasal syndrome or multisystem atrophy | 80% agreed |
| --- | --- |
| Comments:  PSP sometimes have MIBG scan abnormalities. (Springer, PET & SPECT in Neurology, 2020) | |

Following this comment, a statement on PSP was separated from the other two syndromes for Round 2.

**Result from Round 2:**

Cardiac MIBG may be particularly useful:

| When the differential diagnosis includes corticobasal syndrome or multisystem atrophy | 71% agreed |
| --- | --- |

| Cardiac MIBG may be useful when the differential diagnosis includes progressive supranuclear palsy, but abnormalities have been reported in progressive supranuclear palsy | 87% agreed |
| --- | --- |

**Note**

If the Round 2 statement was accepted, there would be an agreed statement on progressive supranuclear palsy but nothing on corticobasal syndrome or multisystem atrophy. The Core Research Group and Delphi Panel agreed to include the statement from Round 1, with a footnote: “Abnormal scans have been reported in clinically diagnosed progressive supranuclear palsy and multiple system atrophy [Catalan et al. 2021 doi: 10.1002/mdc3.1322]”
