# Supplementary material for: Delphi consensus guidelines for the use of striatal dopaminergic imaging and cardiac metaiodobenzylguanidine (MIBG) scintigraphy for the diagnosis of dementia and mild cognitive impairment with Lewy bodies
Source: Alzheimers Dement (Amst). 2026 Mar 4;18(1):e70296. doi: 10.1002/dad2.70296 (PMC12960062; doi:10.1002/dad2.70296)
Supplement: Supplementary file 4 — Supporting Information [file DAD2-18-e70296-s004.docx]

**Delphi Consensus Guidelines for the use of striatal dopaminergic imaging and cardiac** **metaiodobenzylguanidine (MIBG) scintigraphy for the diagnosis of dementia and mild cognitive impairment with Lewy bodies**

**Introduction**

Dementia with Lewy bodies (DLB) and its prodrome, mild cognitive impairment with Lewy bodies (MCI-LB) are common causes of cognitive impairment. DLB and MCI-LB are under-recognised clinically, but clinical diagnosis can be supported through the use of imaging biomarkers, including striatal dopaminergic imaging and cardiac [^123^I]-MIBG scintigraphy (henceforward ‘cardiac MIBG scintigraphy’). These two imaging biomarkers are included as ‘indicative biomarkers’ in the diagnostic criteria for DLB and ‘proposed biomarkers’ for MCI-LB.^[[1]](#footnote-1),^^[[2]](#footnote-2)^ However, there are circumstances where one biomarker may be preferred over the other.

The aim of these Guidelines is to provide clinicians with clear and simple guidance to support the effective use of striatal dopaminergic imaging and cardiac MIBG scintigraphy. The Guidelines are intended for use when DLB and MCI-LB (or other prodromal presentations of DLB) are part of the differential diagnosis. Further information on the diagnosis of DLB and MCI-LB and how striatal dopaminergic imaging and cardiac MIBG scintigraphy are incorporated into diagnostic criteria can be found in the relevant diagnostic guidelines for each condition.^1,2^ Striatal dopaminergic imaging and cardiac MIBG scintigraphy can be used in the diagnosis of Parkinson’s disease (PD) and Parkinson’s disease dementia (PDD), but do not differentiate between MCI-LB, DLB, PD, or PDD.

The method for the development of the Guidelines can be found in the accompanying published paper. The Guidelines are published under a CC-BY license and can be adapted freely to support their use in a range of clinical services (e.g. when only one of the imaging modalities is available).

The Guidelines must be used in conjunction with other relevant information, including the Summary Product Characteristics of the imaging ligands. The information within the Guidelines is specific to the issues faced when assessing a person with cognitive impairment and does not cover generic issues that may affect scan choice (e.g. previous sensitivity to iodine).

Medication half-life data can be found at ‘DailyMed’, which is managed by the National Institutes of Health (USA): <https://dailymed.nlm.nih.gov/dailymed/index.cfm>

The majority of the recommendations are based on expert consensus, with limited direct evidence. However, there has been a significant amount of research investigating the sensitivity and specificity of these biomarkers, particularly for the diagnosis of DLB. Therefore, a strength of recommendation was assigned by the authors for the ‘Indications’ section of the Guideline, based on the Strength of Recommendation Taxonomy ([doi.org/10.3122/jabfm.17.1.59](https://doi.org/10.3122/jabfm.17.1.59)). This allows the categorisation of strength of recommendation into three categories:

A – consistent, good quality patient-oriented evidence

B – Inconsistent or limited quality patient-oriented evidence

C – Consensus, disease-oriented evidence, usual practice, expert opinion, or case series for studies of diagnosis, treatment, prevention, or screening

# Indication for use and choice of scan

## Striatal dopaminergic imaging and cardiac MIBG scintigraphy should only be used where the result will have an impact on patient care or quality of life.

## Striatal dopaminergic imaging is indicated in the following situations:

- When the diagnosis is uncertain, and DLB [A]^[[3]](#footnote-3)^ or MCI-LB [B] is suspected
- In late onset (>60 years) psychiatric disorders (without MCI) where Lewy body disease is a suspected cause [C]
- In recurrent, prolonged or unexplained delirium (without MCI), where Lewy body disease is a suspected cause [C]

## Cardiac MIBG scintigraphy is indicated in the following situations:

- When the diagnosis is uncertain, and DLB [A] or MCI-LB [B] is suspected

## When striatal dopaminergic imaging and cardiac MIBG are both available, dopaminergic imaging should be the first-choice investigation in most cases. [C]

## If striatal dopaminergic imaging is normal, but DLB or MCI-LB is still suspected, cardiac MIBG scintigraphy is an appropriate investigation. If cardiac MIBG scintigraphy is normal, but DLB or MCI-LB is still suspected, striatal dopaminergic imaging is an appropriate investigation. [C]

## If striatal dopaminergic imaging and/or cardiac MIBG scintigraphy is normal, repeat imaging in the same modality should only be undertaken if there has been significant clinical progression, and the diagnosis remains uncertain.^[[4]](#footnote-4)^ [C]

# Striatal dopaminergic imaging

## Striatal dopaminergic imaging:

- is particularly useful in cases where parkinsonism is suspected, but not certain clinically [C]
- is particularly useful to differentiate parkinsonism due to DLB or MCI-LB from drug-induced parkinsonism [B].
- may be useful to differentiate parkinsonism due to DLB or MCI-LB from vascular parkinsonism^[[5]](#footnote-5)^ [B]
- should be interpreted alongside recent structural imaging (i.e. CT or MRI) [C]
- may be abnormal in frontotemporal dementia [B]
- should not be used to differentiate DLB or MCI-LB from progressive supranuclear palsy, corticobasal syndrome or multiple system atrophy. [A]

The following statements refer specifically to [^123^I]-FP-CIT SPECT, the most widely used ligand for striatal dopamine transporter imaging.

## The following medications and recreational drugs should be stopped for five half-lives before undertaking striatal dopaminergic imaging using [^123^I]-FP-CIT SPECT [C]:^[[6]](#footnote-6)^

- Cocaine, Amphetamines
- Methylphenidate, Bupropion, Radafaxine
- Modafinil
- Ephedrine and Phenteramine

## The use of the following drugs should be noted when interpreting [^123^I]-FP-CIT SPECT [C]:

- Lithium (may decrease signal)
- Selective serotonin reuptake inhibitors (may increase signal)^[[7]](#footnote-7),^^[[8]](#footnote-8)^

## Drugs should only be stopped in consultation with the patient and their clinical team, with consideration of the risks of temporarily stopping medications and the likelihood of the medication significantly affecting the scan result. [C]

## Dopaminergic imaging abnormalities caused by medications would be expected to cause balanced loss. Evidence of regional loss (e.g. in one putamen) is more suggestive of striatal dopamine transporter loss associated with neurodegeneration. [C]

# Cardiac MIBG scintigraphy

## Cardiac MIBG scintigraphy may be particularly useful:

- In patients that are unable to complete SPECT imaging of the head (e.g. because of claustrophobia or inability to keep head still) [C]
- In patients with an indeterminate/borderline result on striatal dopaminergic imaging [C]
- When the differential diagnosis includes progressive supranuclear palsy, corticobasal syndrome or multiple system atrophy^[[9]](#footnote-9)^ [C]

## Cardiac MIBG scintigraphy should not be used to help diagnose DLB or MCI-LB in people with:

- Heart failure – New York Heart Classification Class II and above (mild shortness of breath and/or angina and slight limitation during ordinary activity) [A]
- Autonomic neuropathy (including diabetic autonomic neuropathy) [B]
- Diabetes with end organ damage (e.g. retinopathy, nephropathy, peripheral neuropathy) [B]
- A history of recent myocardial infarction (past 12 months) [B]

## When using cardiac MIBG scintigraphy, conclusions about the presence of Lewy body disease should be made with caution in people with:

- A history of myocardial infarction [B]
- Diabetes [B]

## For patients with diabetes, information on the duration of diabetes, severity and disease control (including medication taken) should be considered by those requesting and interpreting the cardiac MIBG scintigraphy result. [C]

## The following medications and recreational drugs should be stopped for five half-lives before undertaking cardiac MIBG scintigraphy as they may reduce the heart:mediastinum ratio [C]:

- Tricyclic antidepressants
- Labetalol
- Reserpine/guanethidine/bretylium
- Sympathomimetics and decongestants (e.g. phenylpropanolamine, ephedrine, pseudoephedrine, phenylephrine, isoproterenol, terbutaline, phenoterol, xylometazoline)
- Cocaine, amphetamine
- Methylphenidate

## Consideration should be given to stopping the following medications prior to imaging. If they are not stopped, they should be taken into account when interpreting cardiac MIBG imaging^[[10]](#footnote-10)^ [C]

- Noradrenaline and serotonin/noradrenaline reuptake inhibitors (SNRIs)
- Tramadol, methadone, pethidine, dextromethorphan, fentanyl, tapentadol^[[11]](#footnote-11)^

## EANM/EANC guidelines recommend stopping first-generation antipsychotics prior to imaging. There is uncertainty about their effect on cardiac MIBG, but in any case, first-generation antipsychotics should generally be avoided in people with suspected DLB or MCI-LB. [C]

## Medications should only be stopped in consultation with the patient and their clinical team, with consideration of the risks of temporarily stopping medications and the likelihood of the medication significantly affecting the scan result. [C]

**Legal Disclaimer**

All the information and advice within this guideline has been assembled to the best of our knowledge. The guideline is intended for information and use by healthcare professionals only and not intended to be a substitute for independent professional medical advice, diagnosis or treatment. Information and advice provided in the guideline does not override the responsibility of healthcare professionals to make decisions appropriate to the circumstances of each patient, in consultation with the patient and/or their guardian or carer. Health care professional users of the guideline are wholly responsible for the care of their patient’s medical care, treatment and oversight. Although the greatest possible care has been taken in compiling the guideline, we cannot guarantee that the information provided within the guideline is accurate. Newcastle University and guideline authors accept no liability for the accuracy of the contents of the guideline and assume no responsibility for any loss, damage or inconvenience caused as a result of any reliance on the guideline whatsoever.

1. *McKeith et al. 2017 doi: 10.1212/WNL.0000000000004058.* [↑](#footnote-ref-1)
2. *McKeith et al. 2020 doi: 10.1212/WNL.0000000000009323.* [↑](#footnote-ref-2)
3. *Strength of Recommendation Taxonomy, see the Introduction for details. A=consistent good quality patient oriented evidence; B=inconsistent or limited quality patient oriented evidence; C= Consensus, disease-oriented evidence, usual practice, expert opinion, or case series for studies of diagnosis, treatment, prevention, or screening* [↑](#footnote-ref-3)
4. *There was no consensus on the time that should elapse before imaging is repeated* [↑](#footnote-ref-4)
5. *‘may be useful’ is used here to convey lower certainty. This indication did not reach consensus in Round 1 when the terminology of ‘is particularly useful’ was used.*  [↑](#footnote-ref-5)
6. *EANM/SNMMI guidelines [Morbelli et al. 2020 doi:10.1007/s00259-020-04817-8] and/or a recent systematic review [Chahid et al. 2023 doi:10.1007/s00259-023-06171-x] recommend stopping the following drugs for 5 half-lives prior to imaging. The majority of Delphi panel members agreed with this, but the level of agreement did not reach consensus: fentanyl, codeine, ketamine, phencyclidine, isoflurane, cannabidiol, haloperidol, benzatropine.* [↑](#footnote-ref-6)
7. *Serotonin-noradrenaline reuptake inhibitors by definition inhibit serotonin reuptake.* [↑](#footnote-ref-7)
8. *The [^123^I]-FP-CIT Summary of Product Characteristics states that selective serotonin*

   *reuptake inhibitors may increase or decrease binding to the dopamine*

   *transporter.* [↑](#footnote-ref-8)
9. *Abnormal scans have been reported in clinically diagnosed progressive supranuclear palsy and multiple system atrophy [Catalan et al. 2021 doi: 10.1002/mdc3.13227]* [↑](#footnote-ref-9)
10. *EANM/EANC guidelines [Flotats et al. 2010 doi:10.1007/s00259-010-1491-4] recommend stopping the following drugs for 5 half lives prior to imaging. Over 50% of Delphi Panel Members agreed that Cardiac MIBG scintigraphy can be undertaken whilst patients continue to take the following medications, but this did not reach consensus: beta-agonists (e.g. salbutamol), levodopa, calcium channel blockers (may increase signal), trazodone* [↑](#footnote-ref-10)
11. *EANM/EANC Guidelines [Flotats et al. 2010 doi:10.1007/s00259-010-1491-4] recommend stopping all opiates, but opiates not on this list have low affinity for NET [Rickli et al. 2018 doi:* *10.1111/bph.14105]* [↑](#footnote-ref-11)
