# Supplementary material for: Delphi consensus guidelines for the use of striatal dopaminergic imaging and cardiac metaiodobenzylguanidine (MIBG) scintigraphy for the diagnosis of dementia and mild cognitive impairment with Lewy bodies
Source: Alzheimers Dement (Amst). 2026 Mar 4;18(1):e70296. doi: 10.1002/dad2.70296 (PMC12960062; doi:10.1002/dad2.70296)
Supplement: Supplementary file 5 — Supporting Information [file DAD2-18-e70296-s003.pdf]

ICMJE DISCLOSURE FORM

Date: 10/6/2025

Your Name: DLB Indicative Imaging Biomarker Study Group

Manuscript Title: Delphi Consensus Guidelines for the use of striatal dopaminergic imaging and cardiac metaiodobenzylguanidine (MIBG) scintigraphy for the diagnosis of dementia and mild cognitive impairment with Lewy bodies

Manuscript Number (if known): TRCI-D-25-00068R1

In the interest of transparency, we ask you to disclose all relationships/activities/interests listed below that are related to the content of your manuscript. "Related" means any relation with for-profit or not-for-profit third parties whose interests may be affected by the content of the manuscript. Disclosure represents a commitment to transparency and does not necessarily indicate a bias. If you are in doubt about whether to list a relationship/activity/interest, it is preferable that you do so.

The author's relationships/activities/interests should be defined broadly. For example, if your manuscript pertains to the epidemiology of hypertension, you should declare all relationships with manufacturers of antihypertensive medication, even if that medication is not mentioned in the manuscript.

In item #1 below, report all support for the work reported in this manuscript without time limit. For all other items, the time frame for disclosure is the past 36 months.

		**Name all entities with whom you have this relationship or indicate none (add rows as needed)**	**Specifications/Comments (e.g., if payments were made to you or to your institution)**
Time frame: Since the initial planning of the work			
1	All support for the present manuscript (e.g., funding, provision of study materials, medical writing, article processing charges, etc.) **No time limit for this item.**	☐ None	
		Instituto de Salud Carlos III	PI18/00435, PI22/00611, INT19/00016, INT23/00048
		Department of Health Generalitat de Catalunya PERIS program	SLT006/17/125
		Intramural Research Program of the National Institutes of Health	C ZIANS003154lick the tab key to add additional rows.
		Wellcome	Wellcome Career Development Award #225263/Z/22/Z Fellowship funding to RW
		Rosetrees and Stoneygate Charitable Trust	
		National Institute for Health and Care Research University College London Hospitals Biomedical Research Centre	
		Parkinson's UK	
		Ann and Billy Harrison Centennial Chair	
		National Institute of Health grants: K08AG052648, P01AG019724, P30AG062422, U01AG057195, U19AG063911, RF1NS050915, R01AG086501, P20-07002.	
		NIH	To the institution
		NIHR Cambridge Biomedical Research Centre	Funding support for the Department of Radiology, University of Cambridge

		Name all entities with whom you have this relationship or indicate none (add rows as needed)	Specifications/Comments (e.g., if payments were made to you or to your institution)
		**Time frame: past 36 months**	
**2**	Grants or contracts from any entity (if not indicated in item #1 above).	☐ **None**	
		Intramural Research Program of the National Institutes of Health	ZIANS003154
		Hersenstichting	payments to institution
		ZonMW	Payments to institution
		Support from National Brain Appeal	
		Parkinson's UK<br>Lewy Body Society	
		Eisai, AriBio, Suven, Biogen, Alzheimer's Association, Cognition Therapeutics, Novo Nordisk, National Institutes of Health	Institutional support for clinical trial participation (no individual compensation)
		NIH/NIA R21AG074368	Paid to institution
		Lewy Body Dementia Association Research Center of Excellence	Paid to institution
		NIH	
		**R56AG085576**	
		**R01AG085571**	
		Lewy Body Society	Payment to institution
		Alzheimer's Research UK	Payment to institution
		National Institute on Aging	Grant funding, payment to institution
		ARISTOS Marie Sklodowska-Curie grant agreement No. 101081134	Payment to institution
		NIH R01AG068128, R01NS121099, P30AG066506, R44AG062072	Payment made to institution
		Florida Department of Health (grants 24A14, 24A15)	Payment made to institution
		Lewy Body Dementia Association Research Center of Excellence program	Payment made to institution
		National Institute for Neurological Research (Programme EXCELES, ID Project No. LX22 NPO5107) – Funded by the European Union – Next Generation EU	Payments made to individual and to institution
		Ministry of Health of the Czech Republic, grant nr. NW25-04-00337	Payments made to individual and to institution
		Czech Science Foundation (GACR) registration number 22-33968S	Payments made to individual and to institution
		Neuron23	Payment to institution for Parkinson's disease clinical trial
		Denali	Payment to institution for Parkinson's disease clinical trial
		Photopharmics/University of Rochester	Payment to institution for Parkinson's disease clinical trial
		NIH	Grant Funding
		Rainwater Charitable<br>Foundation, Blufield Project,<br>Silicon Valley CF.	Payments made to individual.
		Lewy Body Society Project Grant	Institutional grant
		EIP Pharma (now Cervomed)	Transposon Therapeutics

		Name all entities with whom you have this relationship or indicate none (add rows as needed)	Specifications/Comments (e.g., if payments were made to you or to your institution)
		Cognition Therapeutics	North Carolina Dept of Health & Human Services
		Alector Pharma	Kinetix (now PetauriKinect)
		National Institute on Aging P30AG062429	
		AC Immune	Research agreement paid to institution
		National Institute on Aging K23AG073575	institution
		Mangurian-Fixel-McKnight Grant for Pilot studies in Lewy Body dementia	institution
		Includes Wellcome, UKRI and BHF	
		Eisai	Grants (As the chair of the department)
		Sumitomo Pharma	Grants (As the chair of the department)
		Nihon Medi-Physics	Grants (As the chair of the department)
		QuIC-Lewy PHRC	
**3**	Royalties or licenses	☐ **None**	
		Oxford University Press – Textbook	Paid
		**R01AG085571**	
		Cambridge University Press	Royalties
**4**	Consulting fees	☐ **None**	
		Grifols S.A.	
		Lilly	
		Fujirebio-Europe	
		Roche Diagnostics	
		Therakind	
		Accenture	
		Acadia	consultation
		GE Healthcare	consultation
		Newell	consultation
		GLG	consultation
		Takeda	Payment to individual
		BioArtic	Payment to my institution
		NIH R01AG087118	Payment to individual
		Ventyx Biosciences	Consulting fees for study design for Parkinson's disease trial
		Biovie	Rater
		Techspert.io, Putnam Associates, Humanity	Payments made to individual.
		Biogen Inc	To the institution
		Eisai Inc	To the institution
		BioArctic Inc.	To the institution
		Cognition Therapeutics	
		Creative Biopeptides	
		I am a consultant at GE Healthcare	All related payments were to institution.
		GE Healthcare	Payments to individual
		Novo Nordisk	Payments made to institution
		Eli Lilly	Payments made to institution

		Name all entities with whom you have this relationship or indicate none (add rows as needed)	Specifications/Comments (e.g., if payments were made to you or to your institution)
5	Payment or honoraria for lectures, presentations, speakers bureaus, manuscript writing or educational events	☐ None	
		Fujirebio-Europe	
		Roche DIagnostics	
		Nutricia	
		Krka Farmacéutica SL	
		Zambon SAU	
		Esteve Pharmaceuticals	
		Neuraxpharm	
		Alter	
		Lilly	
		GE Healthcare	
		Britannia	
		Bial<br>Shirley Ryan Ability Lab<br>Omnix Pharma	
		American Academy of Clinical Neuropsychology Conference Honorarium	Paid
		University of Tennesee Annual Symposium on Alzheimers and Dementia Honorarium	Paid
		Eisai	Lecture – payment to individual
		6th Taiwan International Congress of Parkinson's Disease and Movement Disorders [virtual] (11/2022)	Speaker honoraria to individual
		American Academy of Neurology Annual Meeting, April 2023 (Boston, MA)	Speaker honoraria/conference registration to individ
		XXVIII World Congress on Parkinson's Disease and Related Disorders. May 2023 (Chicago, IL)	Speaker honoraria/travel support to individual
		PRIME CME Program: "Ask the Expert: Individualizing Treatment Plans and Providing Patient-Centered Care in Advanced Stage Parkinson's Disease." Live and recorded CME. 8/15/2023.	Speaker honoraria to individual
		XXIX World Congress on Parkinson's Disease and Related Disorders. Lisbon, Portugal. May 2024.	Speaker honoraria/travel support to individual
		Keynote speaker for the Dr. Daniel I. Kaufer Lecture Series at the University of Wisconsin Alzheimer's Disease Research Center [virtual]. June 27, 2024.	Speaker honoraria to individual
		Dementia with Lewy Bodies: Filling the Gaps in Translational and Clinical Research (National Institutes of Health conference co-hosted by NIA and NINDS). Bethesda, MD. November 12, 2024.	Speaker travel support to individual
		Michael J. Fox Foundation NeuroImpact Workshop. New York, NY. November 18, 2024.	Speaker travel support to individual

		Name all entities with whom you have this relationship or indicate none (add rows as needed)	Specifications/Comments (e.g., if payments were made to you or to your institution)
		Peking Union Medical College, Beijing, China	Lecture honorarium
		Honoraria from American Academy of Neurology for manuscript and question writing	To individual
		Merck	Speaker Honoraria on MS Imaging
		East of England Prostate Cancer Forum 2024 (invited talk honorarium)	Bayer Public Limited Company
		Eli Lilly	Eisai
		Otsuka Pharma	Kowa Pharm
		Tsumura	
		Honoraria for presentations: Roche, Biogen	
**6**	Payment for expert testimony	☒ **None**	
**7**	Support for attending meetings and/or travel	☐ **None**	
		Fujirebio-Europe	
		Lilly	
		Nutricia	
		Novo Nordisk	
		Wellcome (as part of Wellcome Career Development Award)	
		Invited speaker to European Academy of Neurology 2025	
		Alzheimer's Association	Meeting registration and travel in exchange for speaking at conference
		LBDA	Research Center of Excellence Meeting
		Alzheimer's Research UK	Payment to institution
		Lewy Body Dementia Association	Travel to annual RCOE meeting, payment to institution
		Peking Union Medical College, Beijing, China	Travel expenses
		Kinetix (now PetauriKinect)	
		Lilly	
**8**	Patents planned, issued or pending	☐ **None**	
		WO2019175379 A1 Markers of synaptopathy in neurodegenerative disease	
**9**	Participation on a Data Safety Monitoring Board or Advisory Board	☐ **None**	
		Grifols S.A.	
		Lilly	
		Fujirebio-Europe	

		Name all entities with whom you have this relationship or indicate none (add rows as needed)	Specifications/Comments (e.g., if payments were made to you or to your institution)
		Roche Diagnostics	
		Scientific Advisory Board: Advanced Brain Health Study	
		EISAI	To individual, speaker Bureau
		Efficient	To individual, recording of training module
		ELI LILLY S.P.A.	PERSONAL
		Lewy Body Society	Specialist Advisory Committee
		Alzheimer's Therapeutic Research Institute/Alzheimer's Clinical Trial Consortium	Payment to individual
		Alzheimer's Disease Cooperative Study	Payment to individual
		R01AG083828	Payment to individual
		AviadoBio Pharma	
		Release Therapeutics	Payments made to institution
		Advisory board for Eisai and Novo-Nordisk	
**10**	Leadership or fiduciary role in other board, society, committee or advocacy group, paid or unpaid	☐ **None**	
		Lewy Body Dementia Association	Scientific Advisory Board Member
		Mission MSA	Scientific Advisory Board Member
		GCAN Initiative	Scientific Advisory Board Member
		JAMA Neurology	Editorial Board Member
		Journal of Parkinson's Disease	Editorial Board Member
		Member steering committee E-DLB	
		Vice-president ICDLB	
		Co-Director Dementia Theme, Academic Health Science Centre, University College London Partners	
		American Academy of Neurology	Member ex-officio, Board of Directors (unpaid)
		Lewy Body Dementia Association	Biomarker working group
		Houston Area Parkinson Society	Medical Board
		Lewy Body Ireland	Secretary & board member (unpaid)
		Threshold Services	board member (unpaid)
		UCNS Behavioral Neurology & Neuropsychiatry Certification Examination Committee	Unpaid
		Lewy Body Dementia Association	Member of the Board of Directors
		Lewy Body España	Member of the Scientific Committee
		Spanish Neurological Society	Member of the Scientific Committee
		Alzheimer´s Association	Member of the ISTAART Advisory Council
		Lewy Body Dementia Association Scientific Advisory Board Executive Committee	Unpaid
		Dementia Alliance of North Carolina	
		Board of Directors for the Florida Society of Neurology	unpaid
		RCR CRAI Faculty	Voluntary role [The Royal College of Radiologists (UK)]
		Japan Psychogeriatric Society	President
		COMOP of fondation Alzheimer	

		Name all entities with whom you have this relationship or indicate none (add rows as needed)	Specifications/Comments (e.g., if payments were made to you or to your institution)
**11**	Stock or stock options	☒ **None**	
**12**	Receipt of equipment, materials, drugs, medical writing, gifts or other services	☐ **None**	
		Eli Lilly	Research material support
**13**	Other financial or non-financial interests	☐ **None**	
		Cerevel Therapeutics	Research Support
		A Randomized Placebo-controlled Trial of Zoledronic Acid for Prevention of Fractures in Patients with PD] this was a study site investigator	Institution
		Otsuka Pharma	Advisory Board
		Novo Nordic	Advisory Board
		Ono Pharma	Advisory Board
		Eisai	Advisory Board
		Sumitomo Pharma	Advisory Board
		Eli Lilly	Advisory Board

Please place an "X" next to the following statement to indicate your agreement:

☒ I certify that I have answered every question and have not altered the wording of any of the questions on this form.

ICMJE DISCLOSURE FORM

**Date:**	10/6/2025
**Your Name:**	Alan Thomas
**Manuscript Title:**	Delphi Consensus Guidelines for the use of striatal dopaminergic imaging and cardiac metaiodobenzylguanidine (MIBG) scintigraphy for the diagnosis of dementia and mild cognitive impairment with Lewy bodies
**Manuscript Number (if known):**	DADM-D-25-00260

In the interest of transparency, we ask you to disclose all relationships/activities/interests listed below that are related to the content of your manuscript. "Related" means any relation with for-profit or not-for-profit third parties whose interests may be affected by the content of the manuscript. Disclosure represents a commitment to transparency and does not necessarily indicate a bias. If you are in doubt about whether to list a relationship/activity/interest, it is preferable that you do so.

The author's relationships/activities/interests should be defined broadly. For example, if your manuscript pertains to the epidemiology of hypertension, you should declare all relationships with manufacturers of antihypertensive medication, even if that medication is not mentioned in the manuscript.

In item #1 below, report all support for the work reported in this manuscript without time limit. For all other items, the time frame for disclosure is the past 36 months.

		**Name all entities with whom you have this relationship or indicate none (add rows as needed)**	**Specifications/Comments (e.g., if payments were made to you or to your institution)**
**Time frame: Since the initial planning of the work**			
1	All support for the present manuscript (e.g., funding, provision of study materials, medical writing, article processing charges, etc.) **No time limit for this item.**	☒ None	
			Click the tab key to add additional rows.
**Time frame: past 36 months**			
**2**	Grants or contracts from any entity (if not indicated in item #1 above).	☒ **None**	
**3**	Royalties or licenses	☒ **None**	

		Name all entities with whom you have this relationship or indicate none (add rows as needed)	Specifications/Comments (e.g., if payments were made to you or to your institution)
**4**	Consulting fees	☒ **None**	
**5**	Payment or honoraria for lectures, presentations, speakers bureaus, manuscript writing or educational events	☒ **None**	
**6**	Payment for expert testimony	☒ **None**	
**7**	Support for attending meetings and/or travel	☒ **None**	
**8**	Patents planned, issued or pending	☒ **None**	
**9**	Participation on a Data Safety Monitoring Board or Advisory Board	☒ **None**	
**10**	Leadership or fiduciary role in other board, society, committee or advocacy group, paid or unpaid	☒ **None**	

		Name all entities with whom you have this relationship or indicate none (add rows as needed)	Specifications/Comments (e.g., if payments were made to you or to your institution)
**11**	Stock or stock options	☒ **None**	
**12**	Receipt of equipment, materials, drugs, medical writing, gifts or other services	☒ **None**	
**13**	Other financial or non-financial interests	☒ **None**	

Please place an "X" next to the following statement to indicate your agreement:

☒ I certify that I have answered every question and have not altered the wording of any of the questions on this form.

ICMJE DISCLOSURE FORM

**Date:**	9/12/2025
**Your Name:**	Gemma Greenfinch
**Manuscript Title:**	Delphi Consensus Guidelines for the use of striatal dopaminergic imaging and cardiac metaiodobenzylguanidine (MIBG) scintigraphy for the diagnosis of dementia and mild cognitive impairment with Lewy bodies
**Manuscript Number (if known):**	DADM-D-25-00260

In the interest of transparency, we ask you to disclose all relationships/activities/interests listed below that are related to the content of your manuscript. "Related" means any relation with for-profit or not-for-profit third parties whose interests may be affected by the content of the manuscript. Disclosure represents a commitment to transparency and does not necessarily indicate a bias. If you are in doubt about whether to list a relationship/activity/interest, it is preferable that you do so.

The author's relationships/activities/interests should be defined broadly. For example, if your manuscript pertains to the epidemiology of hypertension, you should declare all relationships with manufacturers of antihypertensive medication, even if that medication is not mentioned in the manuscript.

In item #1 below, report all support for the work reported in this manuscript without time limit. For all other items, the time frame for disclosure is the past 36 months.

		**Name all entities with whom you have this relationship or indicate none (add rows as needed)**	**Specifications/Comments (e.g., if payments were made to you or to your institution)**
**Time frame: Since the initial planning of the work**			
1	All support for the present manuscript (e.g., funding, provision of study materials, medical writing, article processing charges, etc.) **No time limit for this item.**	☐ None	
		Alzheimer's Society Project Grant	
			Click the tab key to add additional rows.
**Time frame: past 36 months**			
**2**	Grants or contracts from any entity (if not indicated in item #1 above).	☐ **None**	
		MRC Confidence in Concept Grant	
**3**	Royalties or licenses	☒ **None**	

		Name all entities with whom you have this relationship or indicate none (add rows as needed)	Specifications/Comments (e.g., if payments were made to you or to your institution)
**4**	Consulting fees	☒ **None**	
**5**	Payment or honoraria for lectures, presentations, speakers bureaus, manuscript writing or educational events	☐ **None**	
		GE Healthcare, for presentations on the quantification of DaTSCAN dopaminergic imaging	
**6**	Payment for expert testimony	☒ **None**	
**7**	Support for attending meetings and/or travel	☒ **None**	
**8**	Patents planned, issued or pending	☒ **None**	
**9**	Participation on a Data Safety Monitoring Board or Advisory Board	☒ **None**	
**10**	Leadership or fiduciary role in other board, society, committee or advocacy group, paid or unpaid	☒ **None**	

		Name all entities with whom you have this relationship or indicate none (add rows as needed)	Specifications/Comments (e.g., if payments were made to you or to your institution)
**11**	Stock or stock options	☒ **None**	
**12**	Receipt of equipment, materials, drugs, medical writing, gifts or other services	☒ **None**	
**13**	Other financial or non-financial interests	☒ **None**	

Please place an "X" next to the following statement to indicate your agreement:

☒ I certify that I have answered every question and have not altered the wording of any of the questions on this form.

ICMJE DISCLOSURE FORM

**Date:**	9/12/2025
**Your Name:**	George Petrides
**Manuscript Title:**	Delphi Consensus Guidelines for the use of striatal dopaminergic imaging and cardiac metaiodobenzylguanidine (MIBG) scintigraphy for the diagnosis of dementia and mild cognitive impairment with Lewy bodies
**Manuscript Number (if known):**	DADM-D-25-00260

In the interest of transparency, we ask you to disclose all relationships/activities/interests listed below that are related to the content of your manuscript. "Related" means any relation with for-profit or not-for-profit third parties whose interests may be affected by the content of the manuscript. Disclosure represents a commitment to transparency and does not necessarily indicate a bias. If you are in doubt about whether to list a relationship/activity/interest, it is preferable that you do so.

The author's relationships/activities/interests should be defined broadly. For example, if your manuscript pertains to the epidemiology of hypertension, you should declare all relationships with manufacturers of antihypertensive medication, even if that medication is not mentioned in the manuscript.

In item #1 below, report all support for the work reported in this manuscript without time limit. For all other items, the time frame for disclosure is the past 36 months.

		**Name all entities with whom you have this relationship or indicate none (add rows as needed)**	**Specifications/Comments (e.g., if payments were made to you or to your institution)**
**Time frame: Since the initial planning of the work**			
1	All support for the present manuscript (e.g., funding, provision of study materials, medical writing, article processing charges, etc.) **No time limit for this item.**	☒ None	
			Click the tab key to add additional rows.
**Time frame: past 36 months**			
2	Grants or contracts from any entity (if not indicated in item #1 above).	☒ **None**	
3	Royalties or licenses	☒ **None**	

		Name all entities with whom you have this relationship or indicate none (add rows as needed)	Specifications/Comments (e.g., if payments were made to you or to your institution)
**4**	Consulting fees	☒ **None**	
**5**	Payment or honoraria for lectures, presentations, speakers bureaus, manuscript writing or educational events	☐ **None**	
		GE Healthcare	To me for delivering educational workshops on FP-CIT imaging, for FP-CIT clinical reporting, manuscript writing (not current manuscript) and for contributing to GE Healthcare led studies
**6**	Payment for expert testimony	☒ **None**	
**7**	Support for attending meetings and/or travel	☒ **None**	
**8**	Patents planned, issued or pending	☒ **None**	
**9**	Participation on a Data Safety Monitoring Board or Advisory Board	☒ **None**	
**10**	Leadership or fiduciary role in other board, society, committee or advocacy group, paid or unpaid	☒ **None**	

		Name all entities with whom you have this relationship or indicate none (add rows as needed)	Specifications/Comments (e.g., if payments were made to you or to your institution)
**11**	Stock or stock options	☒ **None**	
**12**	Receipt of equipment, materials, drugs, medical writing, gifts or other services	☒ **None**	
**13**	Other financial or non-financial interests	☒ **None**	

Please place an "X" next to the following statement to indicate your agreement:

☒ I certify that I have answered every question and have not altered the wording of any of the questions on this form.

ICMJE DISCLOSURE FORM

**Date:**	9/15/2025
**Your Name:**	Hein J. Verberne
**Manuscript Title:**	Delphi Consensus Guidelines for the use of striatal dopaminergic imaging and cardiac metaiodobenzylguanidine (MIBG) scintigraphy for the diagnosis of dementia and mild cognitive impairment with Lewy bodies
**Manuscript Number (if known):**	DADM-D-25-00260

In the interest of transparency, we ask you to disclose all relationships/activities/interests listed below that are related to the content of your manuscript. "Related" means any relation with for-profit or not-for-profit third parties whose interests may be affected by the content of the manuscript. Disclosure represents a commitment to transparency and does not necessarily indicate a bias. If you are in doubt about whether to list a relationship/activity/interest, it is preferable that you do so.

The author's relationships/activities/interests should be defined broadly. For example, if your manuscript pertains to the epidemiology of hypertension, you should declare all relationships with manufacturers of antihypertensive medication, even if that medication is not mentioned in the manuscript.

In item #1 below, report all support for the work reported in this manuscript without time limit. For all other items, the time frame for disclosure is the past 36 months.

		**Name all entities with whom you have this relationship or indicate none (add rows as needed)**	**Specifications/Comments (e.g., if payments were made to you or to your institution)**
**Time frame: Since the initial planning of the work**			
1	All support for the present manuscript (e.g., funding, provision of study materials, medical writing, article processing charges, etc.) **No time limit for this item.**	☒ None	
			Click the tab key to add additional rows.
**Time frame: past 36 months**			
**2**	Grants or contracts from any entity (if not indicated in item #1 above).	☒ **None**	
**3**	Royalties or licenses	☒ **None**	

		Name all entities with whom you have this relationship or indicate none (add rows as needed)	Specifications/Comments (e.g., if payments were made to you or to your institution)
**4**	Consulting fees	☒ **None**	
**5**	Payment or honoraria for lectures, presentations, speakers bureaus, manuscript writing or educational events	☒ **None**	
**6**	Payment for expert testimony	☒ **None**	
**7**	Support for attending meetings and/or travel	☒ **None**	
**8**	Patents planned, issued or pending	☒ **None**	
**9**	Participation on a Data Safety Monitoring Board or Advisory Board	☒ **None**	
**10**	Leadership or fiduciary role in other board, society, committee or advocacy group, paid or unpaid	☒ **None**	

		Name all entities with whom you have this relationship or indicate none (add rows as needed)	Specifications/Comments (e.g., if payments were made to you or to your institution)
**11**	Stock or stock options	☒ **None**	
**12**	Receipt of equipment, materials, drugs, medical writing, gifts or other services	☒ **None**	
**13**	Other financial or non-financial interests	☒ **None**	

Please place an "X" next to the following statement to indicate your agreement:

☒ I certify that I have answered every question and have not altered the wording of any of the questions on this form.

ICMJE DISCLOSURE FORM

**Date:**	9/21/2025
**Your Name:**	Joseph Kane
**Manuscript Title:**	Delphi Consensus Guidelines for the use of striatal dopaminergic imaging and cardiac metaiodobenzylguanidine (MIBG) scintigraphy for the diagnosis of dementia and mild cognitive impairment with Lewy bodies
**Manuscript Number (if known):**	DADM-D-25-00260

In the interest of transparency, we ask you to disclose all relationships/activities/interests listed below that are related to the content of your manuscript. "Related" means any relation with for-profit or not-for-profit third parties whose interests may be affected by the content of the manuscript. Disclosure represents a commitment to transparency and does not necessarily indicate a bias. If you are in doubt about whether to list a relationship/activity/interest, it is preferable that you do so.

The author's relationships/activities/interests should be defined broadly. For example, if your manuscript pertains to the epidemiology of hypertension, you should declare all relationships with manufacturers of antihypertensive medication, even if that medication is not mentioned in the manuscript.

In item #1 below, report all support for the work reported in this manuscript without time limit. For all other items, the time frame for disclosure is the past 36 months.

		**Name all entities with whom you have this relationship or indicate none (add rows as needed)**	**Specifications/Comments (e.g., if payments were made to you or to your institution)**
**Time frame: Since the initial planning of the work**			
1	All support for the present manuscript (e.g., funding, provision of study materials, medical writing, article processing charges, etc.) **No time limit for this item.**	☒ None	
			Click the tab key to add additional rows.
**Time frame: past 36 months**			
**2**	Grants or contracts from any entity (if not indicated in item #1 above).	☐ **None**	
		Lewy Body Society Project Grant	
**3**	Royalties or licenses	☒ **None**	

		Name all entities with whom you have this relationship or indicate none (add rows as needed)	Specifications/Comments (e.g., if payments were made to you or to your institution)
**4**	Consulting fees	☒ **None**	
**5**	Payment or honoraria for lectures, presentations, speakers bureaus, manuscript writing or educational events	☐ **None**	
		Lewy Body Academy	Paid to me
**6**	Payment for expert testimony	☒ **None**	
**7**	Support for attending meetings and/or travel	☐ **None**	
		Alzheimer's Research UK travel grant	Paid to my institution – contribution towards travel to ILBDC to present this work
**8**	Patents planned, issued or pending	☒ **None**	
**9**	Participation on a Data Safety Monitoring Board or Advisory Board	☐ **None**	
		Lewy Body Society	Unpaid member of Scientific Advisory Committee
**10**	Leadership or fiduciary role in other board, society, committee or advocacy group, paid or unpaid	☐ **None**	
		Lewy Body Ireland	Unpaid board member and secretary

		Name all entities with whom you have this relationship or indicate none (add rows as needed)	Specifications/Comments (e.g., if payments were made to you or to your institution)
**11**	Stock or stock options	☒ **None**	
**12**	Receipt of equipment, materials, drugs, medical writing, gifts or other services	☒ **None**	
**13**	Other financial or non-financial interests	☒ **None**	

Please place an "X" next to the following statement to indicate your agreement:

☒ I certify that I have answered every question and have not altered the wording of any of the questions on this form.

ICMJE DISCLOSURE FORM

**Date:**	10/9/2025
**Your Name:**	Paul C Donaghy
**Manuscript Title:**	Delphi Consensus Guidelines for the use of striatal dopaminergic imaging and cardiac metaiodobenzylguanidine (MIBG) scintigraphy for the diagnosis of dementia and mild cognitive impairment with Lewy bodies
**Manuscript Number (if known):**	DADM-D-25-00260

In the interest of transparency, we ask you to disclose all relationships/activities/interests listed below that are related to the content of your manuscript. "Related" means any relation with for-profit or not-for-profit third parties whose interests may be affected by the content of the manuscript. Disclosure represents a commitment to transparency and does not necessarily indicate a bias. If you are in doubt about whether to list a relationship/activity/interest, it is preferable that you do so.

The author's relationships/activities/interests should be defined broadly. For example, if your manuscript pertains to the epidemiology of hypertension, you should declare all relationships with manufacturers of antihypertensive medication, even if that medication is not mentioned in the manuscript.

In item #1 below, report all support for the work reported in this manuscript without time limit. For all other items, the time frame for disclosure is the past 36 months.

		**Name all entities with whom you have this relationship or indicate none (add rows as needed)**	**Specifications/Comments (e.g., if payments were made to you or to your institution)**
		Time frame: Since the initial planning of the work	
1	All support for the present manuscript (e.g., funding, provision of study materials, medical writing, article processing charges, etc.) **No time limit for this item.**	☐ None	
		Medical Research Council	Institution
			Click the tab key to add additional rows.
		Time frame: past 36 months	
2	Grants or contracts from any entity (if not indicated in item #1 above).	☐ **None**	
		Alzheimer's Society	Institution
		Michael J Fox Foundation	Institution
		NIHR Newcastle Biomedical Research Centre	Institution
		Alzheimer's Research UK	Institution
		GE Healthcare	Institution

		Name all entities with whom you have this relationship or indicate none (add rows as needed)	Specifications/Comments (e.g., if payments were made to you or to your institution)
3	Royalties or licenses	☒ None	
4	Consulting fees	☒ None	
5	Payment or honoraria for lectures, presentations, speakers bureaus, manuscript writing or educational events	☐ None	
		Neurology Academy	Institution
6	Payment for expert testimony	☒ None	
7	Support for attending meetings and/or travel	☒ None	
8	Patents planned, issued or pending	☒ None	
9	Participation on a Data Safety Monitoring Board or Advisory Board	☒ None	
10	Leadership or fiduciary role in other board,	☐ None	
		Lewy Body Society Specialist Advisory Committee	Unpaid

		Name all entities with whom you have this relationship or indicate none (add rows as needed)	Specifications/Comments (e.g., if payments were made to you or to your institution)
	society, committee or advocacy group, paid or unpaid	Associate Editor Alzheimer's Research and Therapy	Institution
**11**	Stock or stock options	☒ **None**	
**12**	Receipt of equipment, materials, drugs, medical writing, gifts or other services	☒ **None**	
**13**	Other financial or non-financial interests	☒ **None**	

Please place an "X" next to the following statement to indicate your agreement:

☒ I certify that I have answered every question and have not altered the wording of any of the questions on this form.

ICMJE DISCLOSURE FORM

**Date:**	9/15/2025
**Your Name:**	John O'Brien
**Manuscript Title:**	Delphi Consensus Guidelines for the use of striatal dopaminergic imaging and cardiac metaiodobenzylguanidine (MIBG) scintigraphy for the diagnosis of dementia and mild cognitive impairment with Lewy bodies
**Manuscript Number (if known):**	DADM-D-25-00260

In the interest of transparency, we ask you to disclose all relationships/activities/interests listed below that are related to the content of your manuscript. "Related" means any relation with for-profit or not-for-profit third parties whose interests may be affected by the content of the manuscript. Disclosure represents a commitment to transparency and does not necessarily indicate a bias. If you are in doubt about whether to list a relationship/activity/interest, it is preferable that you do so.

The author's relationships/activities/interests should be defined broadly. For example, if your manuscript pertains to the epidemiology of hypertension, you should declare all relationships with manufacturers of antihypertensive medication, even if that medication is not mentioned in the manuscript.

In item #1 below, report all support for the work reported in this manuscript without time limit. For all other items, the time frame for disclosure is the past 36 months.

		**Name all entities with whom you have this relationship or indicate none (add rows as needed)**	**Specifications/Comments (e.g., if payments were made to you or to your institution)**
**Time frame: Since the initial planning of the work**			
1	All support for the present manuscript (e.g., funding, provision of study materials, medical writing, article processing charges, etc.) **No time limit for this item.**	☒ None	
			Click the tab key to add additional rows.
**Time frame: past 36 months**			
**2**	Grants or contracts from any entity (if not indicated in item #1 above).	☒ **None**	
**3**	Royalties or licenses	☒ **None**	

		Name all entities with whom you have this relationship or indicate none (add rows as needed)	Specifications/Comments (e.g., if payments were made to you or to your institution)
**4**	Consulting fees	☐ **None**	
		Biogen	Acted as Consultant
		Roche	Acted as Consultant
		GE Healthcare	Acted as Consultant
		Okwin	Acted as Consultant
**5**	Payment or honoraria for lectures, presentations, speakers bureaus, manuscript writing or educational events	☐ **None**	
		GE Healthcare	Received honorarium for lectures
**6**	Payment for expert testimony	☒ **None**	
**7**	Support for attending meetings and/or travel	☒ **None**	
**8**	Patents planned, issued or pending	☒ **None**	
**9**	Participation on a Data Safety Monitoring Board or Advisory Board	☐ **None**	
		DSMB	Advisory board
		TauRx	Advisory board
		Novo Nordisk	Advisory board
**10**	Leadership or fiduciary role in other board, society, committee or advocacy group, paid or unpaid	☐ **None**	
		UK Alzheimer’s Society Research Strategy Council	Chairman

		Name all entities with whom you have this relationship or indicate none (add rows as needed)	Specifications/Comments (e.g., if payments were made to you or to your institution)
**11**	Stock or stock options	☒ **None**	
**12**	Receipt of equipment, materials, drugs, medical writing, gifts or other services	☒ **None**	
**13**	Other financial or non-financial interests	☐ **None**	
		Avid/ Lilly	Received academic support for research
		Merck	Received academic support for research
		UCB	Received academic support for research
		Alliance Medical	Received academic support for research

Please place an "X" next to the following statement to indicate your agreement:

☒ I certify that I have answered every question and have not altered the wording of any of the questions on this form.

ICMJE DISCLOSURE FORM

Date: 9/25/2025

Your Name: John-Paul Taylor

Manuscript Title: Delphi Consensus Guidelines for the use of striatal dopaminergic imaging and cardiac metaiodobenzylguanidine (MIBG) scintigraphy for the diagnosis of dementia and mild cognitive impairment with Lewy bodies

Manuscript Number (if known): DADM-D-25-00260

In the interest of transparency, we ask you to disclose all relationships/activities/interests listed below that are related to the content of your manuscript. "Related" means any relation with for-profit or not-for-profit third parties whose interests may be affected by the content of the manuscript. Disclosure represents a commitment to transparency and does not necessarily indicate a bias. If you are in doubt about whether to list a relationship/activity/interest, it is preferable that you do so.

The author's relationships/activities/interests should be defined broadly. For example, if your manuscript pertains to the epidemiology of hypertension, you should declare all relationships with manufacturers of antihypertensive medication, even if that medication is not mentioned in the manuscript.

In item #1 below, report all support for the work reported in this manuscript without time limit. For all other items, the time frame for disclosure is the past 36 months.

		**Name all entities with whom you have this relationship or indicate none (add rows as needed)**	**Specifications/Comments (e.g., if payments were made to you or to your institution)**
		**Time frame: Since the initial planning of the work**	
1	All support for the present manuscript (e.g., funding, provision of study materials, medical writing, article processing charges, etc.) **No time limit for this item.**	☐ None	
		National Institute for Health (UK) Biomedical Research Centre via Newcastle University (my employer)	Support for my research and academic activities. Payment to institution.
			Click the tab key to add additional rows.
		**Time frame: past 36 months**	
2	Grants or contracts from any entity (if not indicated in item #1 above).	☒ **None**	
3	Royalties or licenses	☒ **None**	

		Name all entities with whom you have this relationship or indicate none (add rows as needed)	Specifications/Comments (e.g., if payments were made to you or to your institution)
**4**	Consulting fees	☒ **None**	
**5**	Payment or honoraria for lectures, presentations, speakers bureaus, manuscript writing or educational events	☐ **None**	
		GE HealthCare	Lecture Fee, personal
		Bial Pharma	Lecture Fee, personal
		British Psychopharmacology Association	Lecture Fee, personal
**6**	Payment for expert testimony	☒ **None**	
**7**	Support for attending meetings and/or travel	☒ **None**	
**8**	Patents planned, issued or pending	☒ **None**	
**9**	Participation on a Data Safety Monitoring Board or Advisory Board	☒ **None**	
**10**	Leadership or fiduciary role in other board, society, committee or advocacy group, paid or unpaid	☐ **None**	
		Chair of the International Consortium for Dementia with Lewy bodies	unpaid
		Chair of the International Movement Disorders Society Cognition and Lewy body disease Study Group	unpaid

		Name all entities with whom you have this relationship or indicate none (add rows as needed)	Specifications/Comments (e.g., if payments were made to you or to your institution)
**11**	Stock or stock options	☒ **None**	
**12**	Receipt of equipment, materials, drugs, medical writing, gifts or other services	☒ **None**	
**13**	Other financial or non-financial interests	☒ **None**	

Please place an "X" next to the following statement to indicate your agreement:

☒ I certify that I have answered every question and have not altered the wording of any of the questions on this form.
